# Chronic Stress-induced Serotonin Impairs Intestinal Epithelial Cell Mitochondrial Biogenesis via the AMPK-PGC-1α Axis

**DOI:** 10.7150/ijbs.97275

**Published:** 2024-08-19

**Authors:** Ding Yang, Yan Sun, Pei Wen, Yaoxing Chen, Jing Cao, Xuelin Sun, Yulan Dong

**Affiliations:** 1 College of Veterinary Medicine, China Agricultural University, Beijing 100193, P.R. China.; 2 Department of Horticulture and Landscape Architecture, Jiangsu Agri-animal Husbandry Vocational College, Taizhou 225300, P.R. China.; 3 Department of Pharmacy, Beijing Hospital, National Center of Gerontology; Institute of Geriatric Medicine, Chinese Academy of Medical Sciences, Beijing 100730, P.R. China.

**Keywords:** serotonin, chronic stress, intestinal barrier, mitochondrial biogenesis, AMPK-PGC-1α axis, 5-HT_7_ receptor

## Abstract

Chronic stress is closely associated with gastrointestinal disorders. However, the impact of stress-related neurotransmitters such as serotonin (5-hydroxytryptamine, 5-HT) on the intestines under chronic stress conditions remains poorly understood. This study aims to elucidate the mechanisms by which 5-HT affects mitochondrial biogenesis and intestinal barrier integrity during chronic stress. Employing a chronic restraint stress (CRS) mouse model, we observed elevated intestinal 5-HT levels, altered colonic mucosal structure, and disrupted tight junctions. The increase in 5-HT was associated with up-regulated serotonin synthesis enzymes and downregulated serotonin reuptake transporters, indicating an imbalance in serotonin homeostasis imbalance caused by chronic stress. Furthermore, serotonin exacerbated oxidative stress and impaired tight junction protein expression, highlighting its role in promoting intestinal barrier dysfunction. Experiments with cells *in vitro* demonstrated that 5-HT impairs mitochondrial biogenesis by inhibiting the AMPK-PGC-1α axis via 5-HT_7_ receptors and the cAMP-PKA pathway. Pharmacological inhibition of serotonin synthesis or 5-HT_7_ receptors alleviated the intestinal barrier damage caused by 5-HT and chronic stress, restoring mitochondrial biogenesis. These findings provide compelling evidence that serotonin exacerbates chronic stress-induced intestinal barrier disruption by inhibiting the AMPK-PGC-1α axis, paving the way for novel therapeutic interventions targeting the detrimental effects of serotonin on the intestine, particularly under chronic stress conditions.

## Introduction

Chronic stress is a condition that arises from the cumulative, repetitive, or prolonged exposure to various stressors, including social isolation, work-related stress, the responsibility of caring for a spouse with mental illness, low socioeconomic status, etc^1^. Studies in both animal models and human studies subjects have found that chronic stress leads to several adverse effects, including intestinal barrier damage^2^, gastrointestinal motility disorders, dysbiosis of the gut microbiota^3^, dysregulation of immune responses^4^, and the promotion of colorectal tumor progression^5^. These findings are closely associated with gastrointestinal diseases, including irritable bowel syndrome (IBS), inflammatory bowel disease (IBD), and colorectal cancer^6^-^8^. The intestinal barrier, serving as a critical interface between gut microbiota and the immune system, plays a crucial role in maintaining intestinal homeostasis^6^. Damage to the intestinal barrier directly results in increased intestinal permeability, allowing pathogens or endotoxins to enter the bloodstream through the intestinal mucosa, thereby stimulating the immune system and increasing the risk of intestinal infections. Therefore, elucidating understanding the specific mechanisms by which chronic stress disrupts the intestinal barrier is of great significance for the prevention and treatment of intestinal diseases.

Chronic stress activates the sympathetic nervous system and the hypothalamic-pituitary-adrenal (HPA) axis, which leads to the release of various neurotransmitters that may trigger numerous physiological or mental disorders^9^,^10^. Serotonin (5-HT), a neurotransmitter widely expressed in the central nervous system and gastrointestinal tract, is primarily produced by enterochromaffin cells (ECs) in the gut in response to mechanical and various chemical stimuli^11^. Beyond its role in neuroregulation, serotonin participates in various pathophysiological processes, with gut 5-HT implicated in gastrointestinal motility, secretion, inflammation, and epithelial cell development^12^. Excessive 5-HT has been shown to increase susceptibility to experimental colitis and Crohn's disease (CD)^13^ and promote the progression of colitis-associated colorectal cancer^14^. Sushil Chandra Regmi et al. found that serotonin regulates the innate immune response of colon epithelial cells through Nox2-derived reactive oxygen species, and the addition of 5-HT *in vitro* and *in vivo* resulted in reduced expression of E-cadherin in colon epithelial cells^15^, emphasizing the importance of 5-HT in maintaining intestinal barrier function. Furthermore, in mammary epithelial cells, different concentrations of 5-HT have been shown to have opposite effects on regulating transepithelial resistance and the expression of tight junction proteins^16^. Notably, excessive expression of gut 5-HT and increased oxidative stress occur in chronic stress^17^,^18^. However, the intrinsic connection between excessive 5-HT and intestinal oxidative stress in regulating the intestinal barrier remains unclear.

Accumulating evidence suggests that cellular damage is associated with mitochondrial dysfunction^19^. The stability of the intestinal epithelium and the maintenance of tight junctions are key cellular processes that depend on normal mitochondrial function. Mitochondria are highly dynamic organelles that maintain cellular energy production through oxidative phosphorylation, and the cell's energy supply largely depends on the capacity for mitochondrial biogenesis, while oxidative stress is a driving factor of mitochondrial dysfunction. Increased mucosal mitochondrial oxidative phosphorylation (OXPHOS) activity and elevated ATP levels have been shown to protect mice from colitis^20^. Recent reports have confirmed the regulatory role of 5-HT on mitochondrial biogenesis in multiple cell types, including neuronal cells, renal proximal tubule cells, and cardiomyocytes^21^-^23^. However, whether the destructive effect of excessive 5-HT on the intestinal barrier is related to mitochondrial function has not been reported. In this study, we investigated the regulatory role of 5-HT on the intestinal barrier under chronic stress and found that 5-HT damages mitochondrial biogenesis by inhibiting the AMPK/PGC-1α axis, thereby disrupting the integrity of the intestinal barrier.

## Materials and methods

### Animals and treatments

In this study, all experimental methods were performed following the China Agricultural University of Health Guide for the Care and Use of Laboratory Animals, following protocols approved by the Institutional Animal Care and Use Committee of the China Agricultural University, Beijing, China, under permit no. AW11011202-2-1 (Beijing, China). The male C57BL/6 mice (6 to 8 weeks old) were obtained from the Vital River Laboratory Animal Technology Co. Ltd. (Beijing, China). These animals were maintained in an SPF-grade lab individually in cages for 7 days to adapt to the environment, then kept under standardized conditions, including controlled temperature, humidity, and 12-hour light-dark cycles, with ad libitum access to standard chow and tap water.

Chronic restraint stress: Chronic restraint stress (CRS) was performed using previously published protocols^24^. Mice subjected to stress were horizontally restrained within customized 50 mL Falcon tubes equipped with ventilation holes, undergoing daily 6-hour confinement (from 9:00 a.m. to 3:00 p.m.) over a continuous 14-day period. Mice in the control group were left undisturbed for 14 days.

Pcpa treatment: To investigate the impact of 5-HT, the mice in the CRS+Pcpa group were intraperitoneally injected with Pcpa (300 mg/kg, Sigma, dissolved in 0.5% CMC-Na/saline water)^25^ at 8:00 a.m., followed by CRS modeling at 9:00 a.m. Simultaneously, the control and CRS groups received vehicle injections at 8:00 a.m.

Mito-tempo treatment: the mice in the CRS+MT group were intraperitoneally injected with Mito-tempo (2 mg/kg, HY-112879, MCE, dissolved in PBS)^26^ at 8:00 a.m., followed by CRS modeling at 9:00 a.m. Simultaneously, the control and CRS groups received vehicle injections at 8:00 a.m.

SB269970 treatment: To investigate the impact of 5-HT_7_ receptor antagonist, the mice in the CRS+SB269970 group were intraperitoneally injected with SB269970 (30 mg/kg, HY-15370A, MCE, dissolved in PBS)^27^ at 8:00 a.m., followed by CRS modeling at 9:00 a.m. Simultaneously, The control and CRS groups received vehicle injections at 8:00 a.m.

### Fecal composition

Mice were individually placed in bedding-free cages, and fecal pellets were collected over an hour and weighed to obtain their wet weight. These pellets were then dried at 50°C for dry weight determination. From these measurements, the wet-to-dry ratio and fecal water content were calculated.

### Biochemical analysis

NE, CORT, 5-HT, cAMP, and PKA levels in mouse blood or colon tissue were measured using ELISA kits (Meimian Industrial Co., Ltd, Jiangsu, China) following the manufacturer's guidelines. After allowing the strips to warm to room temperature for 20 minutes, standard and sample wells were prepared. Standards (50 μL at varying concentrations) and samples (10 μL) were added to their respective wells, followed by 40 μL of sample diluent for all but the blank wells. Next, 100 μL of HRP-conjugated detection antibody was added to each well, and the plate was incubated at 37°C for 60 minutes. Following incubation, the wells were washed five times and dried. Substrates A and B (50 μL each) were then added, and the plate was incubated again at 37°C for 15 minutes. After adding 50 μL of stop solution, the optical density (OD) at 450 nm was measured within 15 minutes to construct a standard curve and calculate sample concentrations.

### Measurement of oxidative stress

Markers of intestinal antioxidant defense, including Total Antioxidant Capacity (T-AOC), Superoxide Dismutase (SOD), Catalase (CAT), Malondialdehyde (MDA), and the Glutathione/oxidized Glutathione ratio (GSH/GSSG), were measured using commercial assay kits, adhering to the protocols provided by the manufacturer (Nanjing Jiancheng Bioengineering Institute, Nanjing, China). Normalization of the results was based on the protein concentration in each sample. Detection at 450 nm was carried out using a microplate reader (BioTek Co., Ltd., Beijing, China).

### Assessment of intestinal permeability

Intestinal permeability in mice was evaluated using FITC-labeled dextran. Mice, after fasting overnight, received an oral dose of FITC-labeled dextran with a molecular weight of 3000-5000 Da (FD4, 60 mg/100 g body weight, Sigma-Aldrich, Shanghai, China). Serum samples were obtained five hours after administration for the assessment of fluorescence intensity using a microplate reader (BioTek Co., Ltd., Beijing, China). Excitation of the fluorescence occurred at 492 nm, with emission recorded at 525 nm.

### Transmission electron microscopy

To examine mitochondrial morphology in intestinal epithelium cells, freshly harvested colon was immediately fixed in electron microscopy fixation solution at 4°C for 2 to 4 hours. Following a rinse in 0.1 M PBS, the specimens were further fixed in 1% osmium tetroxide at ambient temperature for 2 hours. Subsequent to gradient-based dehydration, the tissues were embedded, sectioned, and then stained with 3% uranyl acetate and lead citrate for 15 minutes. The specimens were visualized and analyzed using a transmission electron microscope (SU3500, Hitachi, Japan). Measurements of mitochondrial count and volume density were conducted with Image J software (National Institutes of Health, Bethesda, MD, USA).

### Cell culture

To investigate the specific mechanism by which serotonin affects intestinal epithelial cells, we conducted in vitro studies using the human colon cancer cell line HT-29 (RRID: CVCL_0320) and Caco-2 cells (RRID: CVCL_0025). The HT-29 cells were cultured in RPMI1640 medium supplemented with 10% FBS, 100 units/mL penicillin, and 0.1 mg/mL streptomycin. The Caco-2 cells were cultured in DMEM supplemented with 20% FBS and 1% penicillin-streptomycin. The cultures were maintained at 37 °C in a humidified incubator with 5% CO_2_. For the experiments, cells were seeded at a density of 1× 10^6^ cells/mL and cultured until they reached confluence. Subsequently, the cells were treated with 5-HT (10 μM, Sigma, USA)^13^ for 24 hours.

For the mitochondrial antioxidant experiments, cells were treated with Mito-tempo (2 μM, HY-112879, MCE, USA)^26^ for 24 hours, both with and without the presence of 5-HT.

In the AMPK-related experiments, cells were pre-treated with the AMPK agonist AICAR (200 μM, MCE, USA)^28^ for 1 hour prior to 24 hours exposure to 5-HT.

For the 5-HTR-related experiments, cells were pre-treatment for 1 hour with the 5-HT_4_ receptor antagonist GR113808 (10 nM, HY-103152, MCE, USA)^29^, the 5-HT_7_ receptor antagonist SB269970 (1 μM, HY-15370A, MCE, USA)^30^, the PKA antagonist H-89 (10 μM, HY-15979A, MCE, USA)^15^, and the c-AMP and PKA agonist Dibutyryl-cAMP (10 μM, dbcAMP, HY-B0764, MCE, USA)^31^. Following pre-treatment, the cells were then exposed to 5-HT for 24 hours.

Following the incubation period, the cultured samples were collected for gene transcript and protein level analyses.

### siRNA interference

HT-29 cells were cultured to 50-60% confluence in culture medium containing no penicillin or streptomycin. HTR7 siRNA (sense: UCACCUUACCUCCACUCUUUGTT; antisense: CAAAGAGUGGAGGUAAGGUGATT), and the negative control siRNA were purchased from Synbio Technologies (Suzhou, China). HT-29 cells were transfected with siRNA using Lipo3000 (L3000015, Invitrogen, Carlsbad, CA, USA) in Opti-MEM (31985062, Gibco, California, USA).

### TER measurement

Confluent HT-29 cell monolayers were cultivated in 24-well transwell chambers (with a polycarbonate membrane, filter pore size of 0.4 μm, and an area of 0.33 cm^2^). Following this, transepithelial electrical resistance (TER) was assessed at 37 °C utilizing an Epithelial Volt Ohm Meter (Millipore, Billerica, MA, USA). The calculation of TER involved the deduction of resistance values attributed to blank filters and the adjustment for the filter's surface area. Measurements were conducted in triplicate to ensure accuracy.

### Mitochondrial function assays

#### Cellular mitochondrial morphology assessment

Live cell mitochondrial morphology was visualized using MitoTracker Deep Red staining (M22426, Invitrogen, CA, USA). Following two PBS washes, cells were incubated with this fluorescent dye at 37°C for 30 minutes, and then examined with a Nikon confocal microscope (Nikon Corporation, Japan). The measurement of mitochondrial length was conducted utilizing ImageJ software (National Institutes of Health, Bethesda, MD, USA).

#### Measurement of intracellular ROS

ROS levels were assessed using flow cytometry and confocal microscopy techniques. For mitochondrial ROS, cells were incubated with MitoSOX (2 µM, Invitrogen, CA, USA) for 30 minutes and washed twice with PBS before analysis by flow cytometry (Beckman, USA). For intracellular ROS, cells were incubated with DCFH-DA (10 μM, MCE, USA) at 37°C for 30 minutes. After incubation, cells were washed with PBS and examined under a confocal microscope (Nikon, Nikon Corporation, Japan).

#### Measurement of mitochondrial membrane potential

The mitochondrial membrane potential was measured using JC-1 (HY-K0601, MCE, USA). According to the instructions, 10 μL of JC-1 (200 μM) was added to achieve a final concentration of 2μM. The cells were incubated at 37 °C in 5% CO_2_ for 15-20 minutes. After incubation, the samples were analyzed with a flow cytometer (Beckman, USA).

#### ATP measurement

ATP levels were measured utilizing an ATP assay kit (S0026, Beyotime Biotechnology, Shanghai, China). In brief, the proximal colon tissues and cell lysates were prepared using the lysis buffer. After centrifugation at 12,000 rpm for 5 minutes at 4°C, the supernatant was retrieved. ATP concentration was assessed by combining 20 μL of this supernatant with 100 μL of the luciferase reagent, which induces luminescence through the reaction of ATP with luciferin, catalyzed by luciferase. Luminescence readings for each sample were obtained using a microplate reader (BioTek Co., Ltd., Beijing, China). Additionally, an ATP standard curve was generated from standards with known concentrations to calibrate the measurements.

#### DNA isolation and mtDNA copy number assay

Quantitative PCR (qPCR) was employed to assess mitochondrial DNA (mtDNA) copy numbers, following methodologies outlined in previous studies^32^,^33^. Total DNA was isolated from either cell cultures or proximal colon samples using the Universal Genomic DNA Extraction Kit (CW2298S, CWBIO, Beijing, China), with 10 ng of extracted DNA utilized for qPCR. In human cells, the mtDNA copy number was quantified by targeting the mitochondrial ND1 gene (mtND1) and normalized against the nuclear gene GAPDH^33^. In mouse samples, quantification was based on the 12S RNA encoded by mitochondria, with normalization to the nuclear ribosomal protein 18S RNA^32^. Primer sequences for the mtDNA quantification are listed in Table S1.

### Histological staining

The proximal colon samples were first washed with PBS, then fixed in a solution of 4% paraformaldehyde, and finally were embedded in paraffin for preservation. Thin sections of the intestinal tissue, measuring 5 μm in thickness, were prepared using a semi-automatic rotary microtome (RM2235, Leica, Germany). Staining procedures, including Hematoxylin and Eosin (H&E) as well as Periodic Acid-Schiff and Alcian Blue (PAS/AB), were applied to these sections to assess tissue morphology.

Immunofluorescence staining was conducted to label proteins such as CgA (Sc-13090, Santa Cruz, Dallas, TX, USA), 5-HT (bs-1126R, Bioss, Beijing, China), MUC2 (ab134119, Abcam, Cambridge, CA, USA), PCNA (10205-2-AP, Abcam, Cambridge, CA, USA), 8-OHdg (bs-1278R, Bioss, Beijing, China), ZO-1 (21773-1-AP, Proteintech, Wuhan, China), and p-PKA (sc-377575, Santa Cruz, Texas, USA) in the tissue sections or cells. The staining process was completed after an overnight incubation at 4°C. This was followed by a rinsing step with PBS on a shaker to remove excess dye. The sections or cells were then incubated with the corresponding secondary antibody at room temperature for 1 hour. The images of tissues or cells were acquired using a Nikon microscope (TE 2000, Nikon Instruments Inc., New York, NY, USA) and evaluated in a blinded manner. The positive staining areas were quantified using Image-Pro Plus software (Media Cybernetics, Rockville, MD, USA).

### Real-Time Quantitative PCR

The proximal colon tissues were obtained from the mice used in the experiment and subjected to homogenization using TRIzol Reagent (CW0580, CoWin Biotech Co., Inc., Beijing, China). The total RNA was then extracted following the guidelines provided by the manufacturer. The RNA sample, weighing 1μg, was subjected to reverse transcription to generate cDNA using the HiScript II Select qRT SuperMix (R312-02, Vazyme, Nanjing, China). The experiment involved the utilization of a real-time quantitative PCR technique, employing gene-specific primer sets and SYBR green master mix (Q141-02, Vazyme, Nanjing, China). The relative expression level of mRNA was detected by Real time system, And fluorescence signals was collected through the FQD 96A system, manufactured by BIOER. Relative mRNA expression levels were calculated with the comparative cycle threshold (Ct; 2^-ΔΔCt^) method. The primers utilized are presented in Table S1.

### Western blot analysis

Protein samples from intestinal tissues and cell lines were lysed using RIPA buffer (R0010, Solarbio Life Sciences, Beijing, China) enriched with protease and phosphatase inhibitors for a duration of 30 minutes. Protein levels were quantified using the BCA Protein Assay Kit, and equal amounts of protein were resolved on 10%-15% SDS-PAGE gels. The proteins were then transferred to a PVDF membrane. This membrane was blocked with 5% skim milk in TBST for 90 minutes at room temperature. Overnight incubation at 4°C with primary antibodies (refer to Table S2) was followed by a 2-hour incubation at room temperature with HRP-linked goat anti-mouse IgG or goat anti-rabbit IgG (Table S2). The immunoblots were imaged and analyzed using Image J software (National Institutes of Health, Bethesda, MD, USA). The intensity of the protein bands was normalized to that of GAPDH.

### Statistical analysis

Data were analyzed with GraphPad Prism version 8.0. Results are shown as mean ± SEM. Data distribution normality was assessed via the Shapiro-Wilk test. Statistical comparisons utilized either the unpaired Student's t-test or one-way ANOVA with Tukey's post-hoc test for multiple comparisons. The data from animal experiments are obtained from at least three biological replicates, whereas the data from cell experiments are derived from three independent experiments to confirm the results. Statistical significance was determined at* P* < 0.05.

## Results

### Chronic restraint stress leads to increased gut 5-HT Levels and changes in related biomarkers in mice

**Figure 1:**
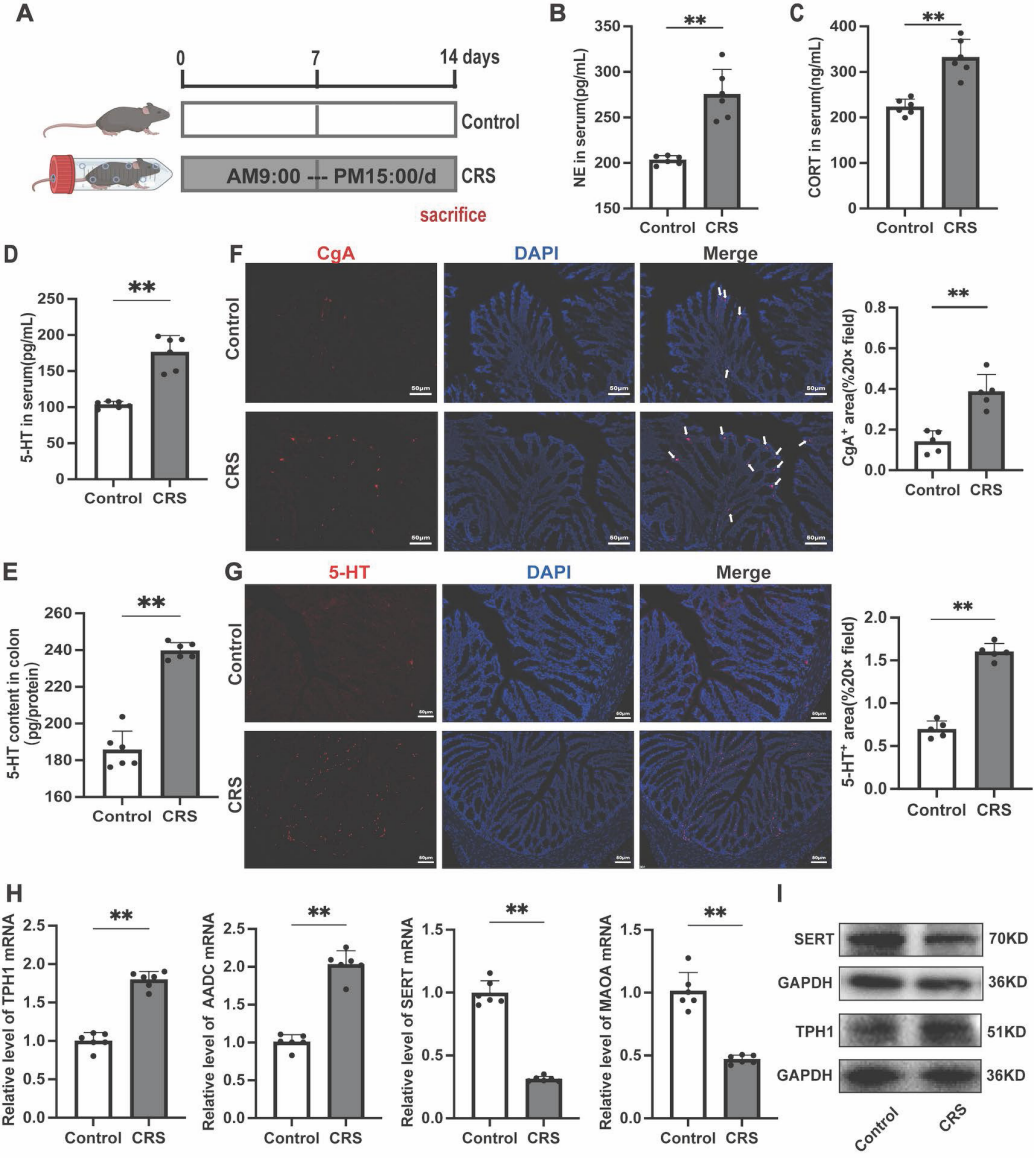
** Impact of Chronic Restraint Stress on the Serotonergic System in Mice.** (A) Diagram of the experimental design for chronic restraint stress (CRS). Serum levels of (B) nor-epinephrine (NE) and (C) corticosterone (CORT) (n = 6). 5-HT levels of (D) Serum and (E) colon tissue (n = 6). (F) Immunofluorescence staining of chromogranin A (CgA, white arrow) in colon sections (scale bar = 100μm), and the area of CgA-positive cells in the colon was quantified. (G) Immunofluorescence staining of 5-HT in the colon (scale bar = 100μm), and the area of 5-HT positive cells was quantified. (H) Gene expression of TPH1, AADC, MAOA and SERT were detected by quantitative PCR (qPCR) (n = 6). (I) The TPH1 and SERT protein expression in the colon. Control: control group; CRS: chronic restraint stress group. Data is presented as the mean ± SEM. *P < 0.05; **P < 0.01.

To investigate the changes in gut 5-HT expression under chronic stress, we established a CRS model based on previous studies (Fig. 1A)^24^. We first assessed the levels of two stress-related hormones, cortisol (CORT) and norepinephrine (NE), and found that both were significantly elevated in the serum of CRS mice (Fig. 1B, C), indicating an activation of the stress response. Serum and colon 5-HT concentrations were significantly higher in CRS mice compared to the control group (Fig. 1D, E). Similar changes were also observed in the small intestine, suggesting a systemic elevation of gut 5-HT levels upon chronic stress exposure (Fig. S1). Immunofluorescence staining revealed significant increases in chromogranin A (CgA) positive cells, the primary sources of gut 5-HT (Fig. 1F), and in the number of 5-HT positive cells within the colon tissue of CRS mice (Fig. 1G). Further examination revealed significant upregulation of the 5-HT bio-synthetic enzymes, tryptophan hydroxylase 1 (TPH1) and aromatic l-amino acid decarboxylase (AADC), as well as a noteworthy downregulation of 5-HT degradative and reuptake mechanisms, involving monoamine oxidase A (MAOA) and the serotonin reuptake transporter (SERT) (Fig. 1H-I). These findings confirm the dysregulation of 5-HT biosynthesis and reuptake under chronic stress conditions, leading to elevated levels of gut serotonin.

### The increase of intestinal 5-HT in chronic stress mice is associated with intestinal dysfunction

**Figure 2:**
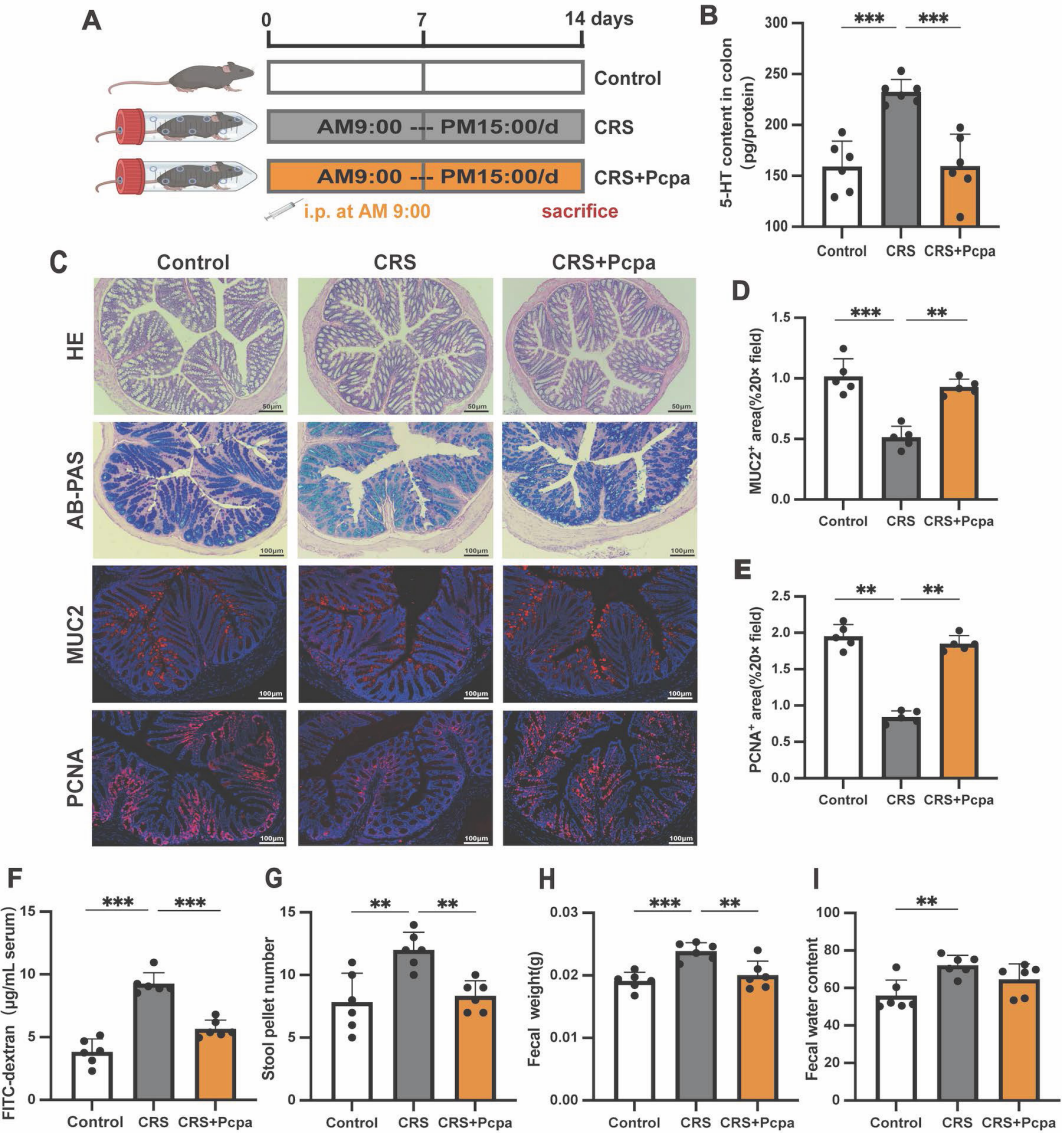
** Elevated Gut 5-HT Linked to Dysfunction in Chronically Stressed Mice.** (A) Intervention and grouping schematic for Pcpa. (B) 5-HT level of colon tissue (n = 6). (C) Representative micrographs showing hematoxylin and eosin (H&E) staining (scale bar = 50 µm), Alcian blue-periodic acid-Schiff (AB-PAS) staining (scale bar = 100 µm), MUC2 immunofluorescence staining (scale bar = 100 µm), and PCNA immunofluorescence staining (scale bar = 100 µm) in the colon of mice. Quantitative analysis of MUC2 expression (D) and PCNA expression (E) in the colon of mice in different groups. (F) Intestinal permeability (n = 6). (G-I) Fecal pellet wet weight and wet: dry ratio of feces measured (n = 6). Control: control group; CRS: chronic restraint stress group; CRS+Pcpa: chronic restraint stress + Pcpa intervention group. Data is presented as the mean ± SEM. *P < 0.05; **P < 0.01; ***P < 0.001.

Recently, the multiple roles of gut 5-HT in intestinal health have attracted widespread attention^13^. To explore the potential effects of chronic stress-induced increases in gut serotonin, we treated CRS mice with the serotonin synthesis inhibitor Pcpa to inhibit gut 5-HT synthesis (Fig. 2A). Pcpa treatment significantly reduced the level of 5-HT in the colon of CRS mice (Fig. 2B). While H&E staining showed no obvious pathological changes in the intestinal structure of the CRS group, but AB-PAS staining revealed a reduction in goblet cells in CRS mice (Fig. 2C). Compared to the control group, the expression of the major mucin component MUC2 and the cell proliferation marker PCNA were significantly reduced in the CRS group (Fig. 2C-E), suggesting that chronic stress may lead to changes in the structure of intestinal epithelial cells. Mice treated with Pcpa exhibited MUC2 expression similar to the control group and increased PCNA expression, indicating that inhibiting 5-HT synthesis could mitigate CRS-induced intestinal changes. Intestinal barrier function was further assessed using the FITC-dextran permeability test, which revealed significant increases in intestinal permeability in the CRS group (Fig. 2F), with improvements observed in the CRS+Pcpa group. Additionally, the number of fecal pellets (Fig. 2G), fecal weight (Fig. 2H), and water content (Fig. 2I) increased in CRS mice increased, indicating a dysfunction in the intestinal barrier of CRS mice. Pcpa treatment appeared partially repair some of the dysfunctions caused by CRS to a certain extent. In summary, our data demonstrate that chronic restraint stress significantly increases gut 5-HT levels in mice, which is associated with gastrointestinal functional disorders.

### Serotonin exacerbates oxidative stress and disrupts tight junctions in chronic stress mice

**Figure 3:**
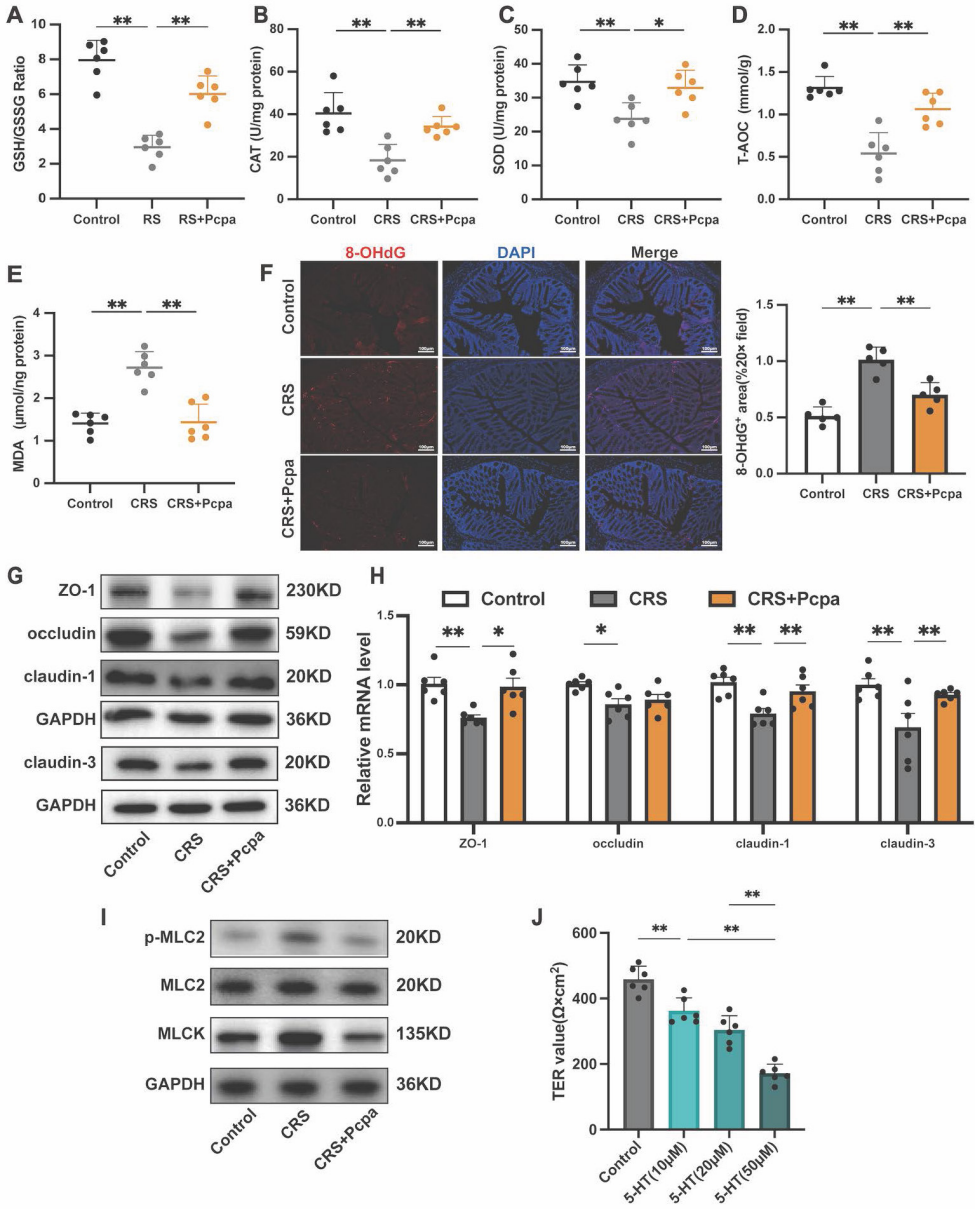
** 5-HT Exacerbates Oxidative Stress and Damages the Colon Barrier.** (A-E) The expression of of GSH/GSSG, CAT, SOD, T-AOC, and MDA of colon (n = 6). (F) Immunofluorescence staining of 8-OHdg in the colon (scale bar = 100μm), and the number of 8-OHdg positive cells was quantified. (G) The protein expression level of ZO-1, occludin, claudin-1, and claudin-3 in the colon. (H) The mRNA levels of ZO-1, occludin, claudin-1, and claudin-3 in the colon (n = 6). (I) The protein expression levels of p-MLC2, MLC2, and MLCK protein in the colon. (J) The TER value of the HT-29 cell monolayers treated with 5-HT (10 μM) for 24 h (n = 6). Control: control group; CRS: chronic restraint stress group; CRS+Pcpa: chronic restraint stress + Pcpa intervention group. Data is presented as the mean ± SEM. *P < 0.05; **P < 0.01.

We further investigated the impact of elevated 5-HT levels on oxidative stress and the intestinal barrier in CRS mice. Measurement of oxidative stress markers revealed a significant reduction in the GSH/GSSG ratio of CRS mice, which was partially reversed by the serotonin synthesis inhibitor Pcpa (Fig. 3A). Dysregulation of antioxidant enzyme activities, such as catalase (CAT) and superoxide dismutase (SOD), indicated an oxidative imbalance (Fig. 3B, C). The total antioxidant capacity (T-AOC) of CRS mice also declined, with Pcpa treatment mitigating this effect (Fig. 3D). CRS group mice showed increased levels of the lipid peroxidation product Malondialdehyde (MDA) and the DNA oxidative damage marker 8-hydroxy-2'-deoxyguanosine (8-OHdG), suggesting a notable rise in tissue oxidative damage. However, Pcpa treatment alleviated this effect (Fig. 3E, F), emphasizing the involvement of 5-HT in exacerbating cellular oxidative stress.

Next, we assessed the integrity of tight junctions, a crucial component of the intestinal barrier. The expression of tight junction proteins, including ZO-1, occludin, claudin-1, and claudin-3, was significantly reduced in the CRS group compared to the control, with Pcpa treatment partially reversing this effect (Fig. 3G, H). These findings underscore the detrimental impact of 5-HT on tight junction integrity during chronic stress. Notably, increased phosphorylation of myosin light chain 2 (MLC2) is known to facilitate the disruption of the tight junction barrier^34^. The expression of phosphorylated MLC2 and myosin light chain kinase (MLCK) was significantly elevated in the CRS group, with these molecular changes also being ameliorated by Pcpa treatment (Fig. 3I), indicating the potential of 5-HT synthesis inhibition in maintaining tight junction integrity.

Finally, to evaluate the impact of 5-HT on the integrity of HT-29 cell layers, we measured the transepithelial electrical resistance (TER). A significant reduction in TER was observed following 5-HT treatment, which was directly proportional to the concentration of 5-HT (Fig. 3J). This further confirms the negative impact of 5-HT on the barrier function of intestinal epithelial cells, suggesting that elevated physiological concentrations may impair intestinal barrier function. In summary, our study results indicate that 5-HT plays a significant role in colon oxidative stress and barrier function damage induced by chronic stress.

### Serotonin compromises mitochondrial biogenesis in the colon of CRS mice

**Figure 4:**
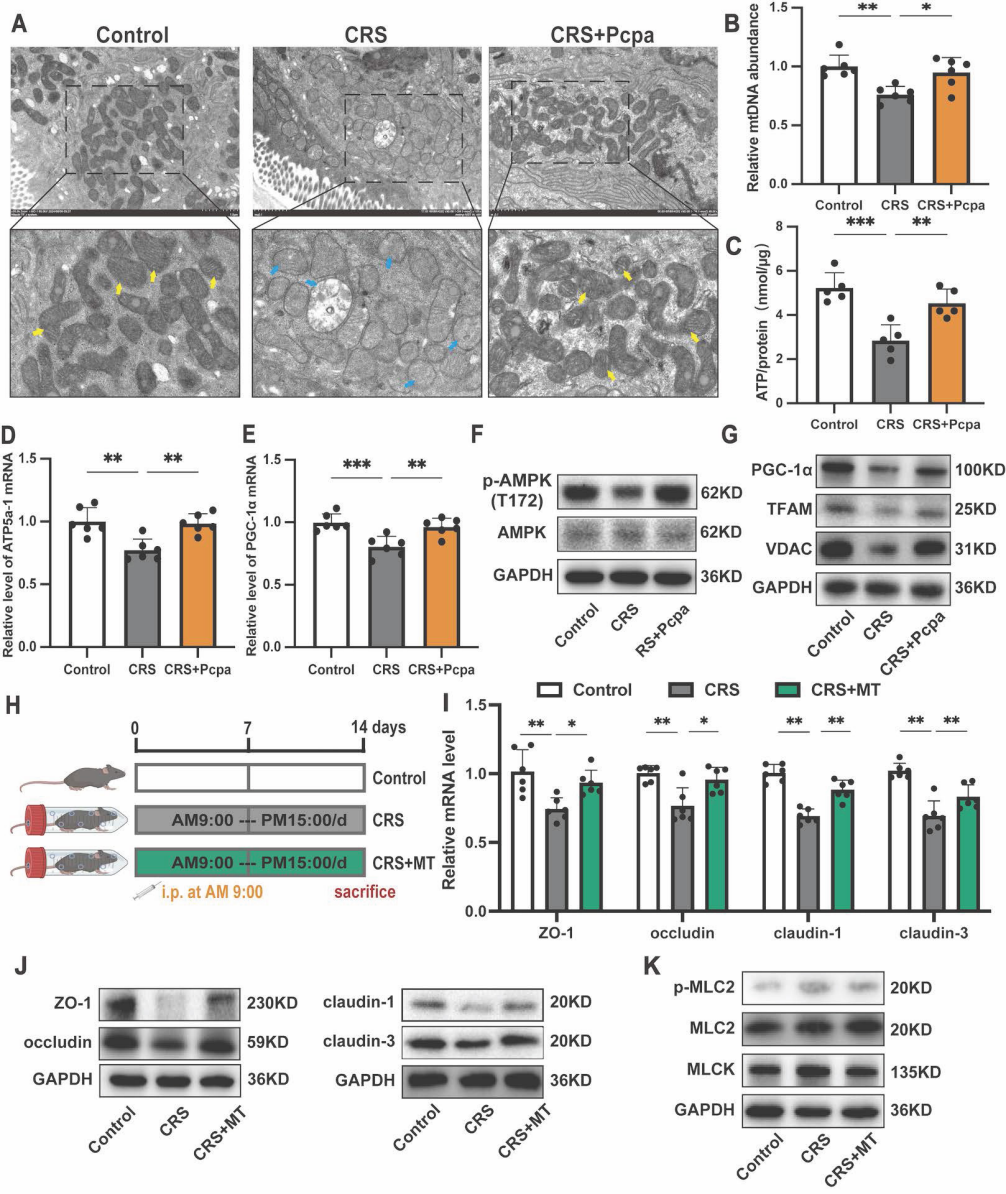
** 5-HT Disrupts Mitochondrial Homeostasis in Colon.** (A) Representative transmission electron microscopy (TEM) images of mitochondria in the colon of mice. The yellow arrows show normal mitochondria and the blue arrows show damaged mitochondria. (B) Measurement of mtDNA levels in the colon (n = 6). (C) Measurement of ATP levels in the colon (n = 4). (D, E) The mRNA expression of ATP5a-1 and PGC-1α in the colon (n = 6). (F) The protein expression of p-AMPK (T172) and AMPK in the colon. (G) The protein expression of PGC-1α, TFAM and VDAC in the colon. (H) Intervention and grouping schematic for Mito-Tempo. (I) The protein expression of ZO-1, occludin, claudin-1, and claudin-3 in the colon. (J) The mRNA levels of ZO-1, occludin, claudin-1, and claudin-3 in the colon (n = 6). (K) The protein expression of p-MLC2, MLC2, and MLCK in the colon. Control: control group; CRS: chronic restraint stress group; CRS+Pcpa: chronic restraint stress + Pcpa intervention group. CRS+MT: chronic restraint stress + Mito-Tempo intervention group. Data is presented as the mean ± SEM. *P < 0.05; **P < 0.01.

Recent studies have highlighted the crucial role of mitochondrial oxidative metabolism in maintaining the integrity of the intestinal epithelial barrier 19. Based on our findings of exacerbated oxidative stress in the colon of mice under chronic stress and the associated increase in 5-HT, we hypothesized that 5-HT might mediate its detrimental effects on tight junction integrity by promoting oxidative stress and mitochondrial dysfunction. Transmission electron microscopy revealed significant mitochondrial damage in intestinal tissues of CRS mice, characterized by mitochondrial swelling, disrupted cristae, and disruption of membrane integrity, which was alleviated by inhibiting 5-HT synthesis (Fig. 4A). Furthermore, mitochondrial DNA (mtDNA) levels (Fig. 4B) and ATP production (Fig. 4C) were significantly reduced in CRS mice but were restored following Pcpa treatment. The expression of genes involved in mitochondrial protein synthesis, ATP5a-1, and peroxisome proliferator-activated receptor gamma coactivator 1-alpha (PGC-1α) decreased in the CRS group. This downregulation was partially reversed by Pcpa treatment (Fig. 4D, E), suggesting that 5-HT might damage mitochondrial biogenesis through oxidative stress pathways.

Western blot analysis showed decreased phosphorylation levels of AMP-activated protein kinase (AMPK), a key regulator of cellular energy homeostasis and mitochondrial biogenesis, in the CRS group, while Pcpa intervention increased p-AMPK (T172) levels (Fig. 4F). Transcription factors vital for mitochondrial biogenesis, such as PGC-1α and mitochondrial transcription factor A (TFAM), exhibited a comparable trend, with decreased expression in CRS mice and increased levels after Pcpa treatment (Fig. 4G). Additionally, the levels of Voltage-Dependent Anion Channels (VDAC), encoded by the nuclear genome of the mitochondria, were reduced (Fig. 4G). These results indicate that 5-HT disrupts mitochondrial biogenesis in the colon of CRS mice. Mitochondrial biogenesis and mitophagy together coordinate mitochondrial homeostasis^35^. We also assessed the levels of autophagy in the colon of CRS mice. Compared to the control group, CRS mice showed significantly reduced levels of LC3-II, PINK1, and Parkin, along with increased accumulation of p62. Pcpa treatment reversed the CRS-induced changes in autophagy-related proteins (Fig. S2A, B), suggesting that 5-HT impairs mitophagy in the colon of CRS mice.

Notably, the use of the mitochondrial antioxidant Mito-Tempo (MT) (Fig. 4H) alleviated the reduction in tight junction protein expression in CRS mice (Fig. 4I, J) and reversed the expression of p-MLC2 and MLCK in CRS (Fig. 4K). This further indicates that 5-HT leads to intestinal barrier dysfunction through mechanisms involving mitochondrial oxidative stress and mitochondrial dysfunction.

### Serotonin interferes with mitochondrial biogenesis and tight junction expression in intestinal epithelial cells

**Figure 5:**
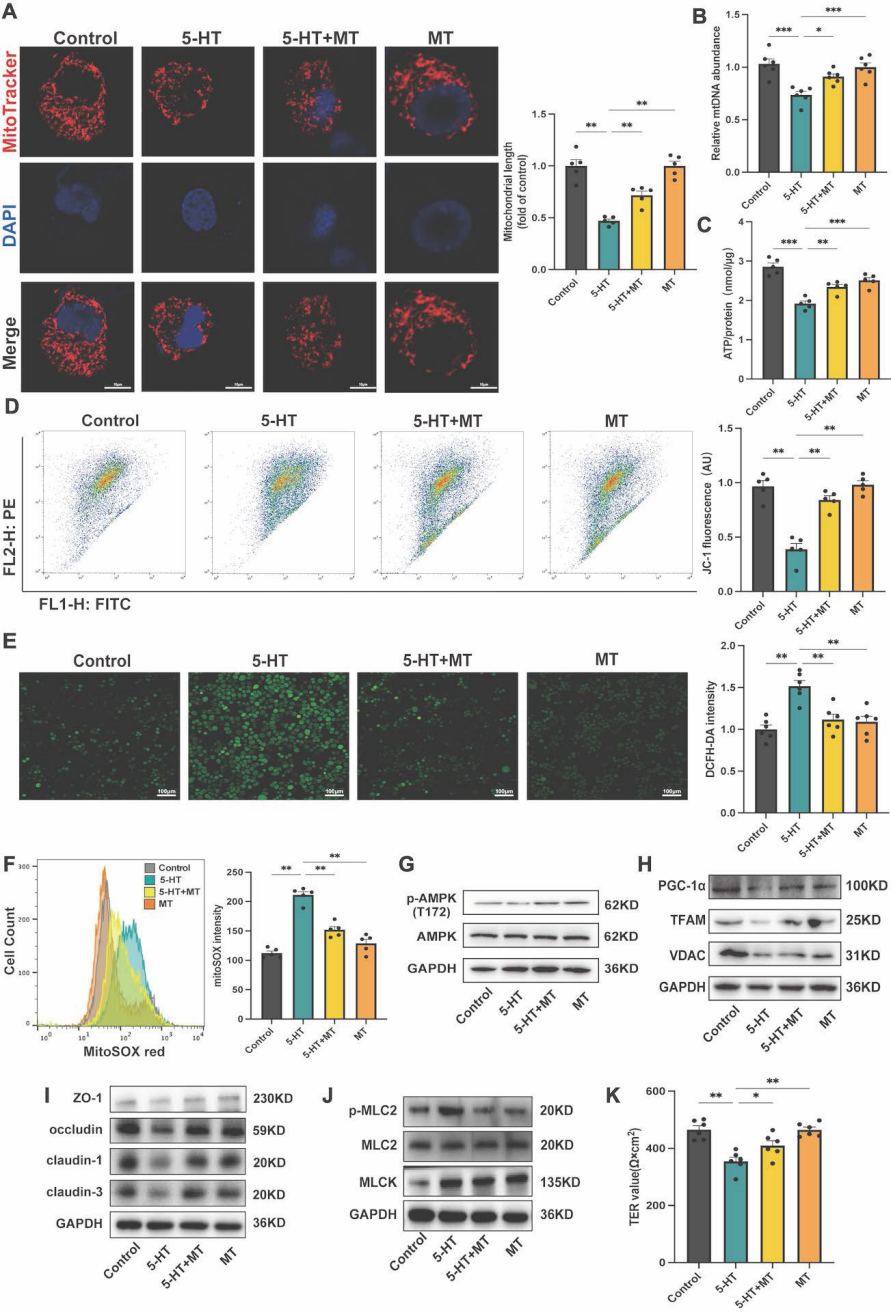
** 5-HT Reduces Tight Junction Expression by Impaction of Mitochondrial Biogenesis in HT-29 Cells.** (A) Representative micrographs showing MitoTracker staining in HT-29 cells treated with 5-HT (10 μM) and Mito-tempo (MT, 10 μM) for 24 h (scale bar = 10 µm) and quantification of mitochondrial length. (B) Measurement of mtDNA levels of HT-29 cells (n = 6). (C) Measurement of ATP levels of HT-29 cells (n = 5). (D) Measurement of mitochondrial membrane potential by JC-1 probes. (E) Determination of ROS levels in HT-29 cells by DCFH-DA fluorescent staining. (F) Measurement of mtROS using mitoSOX staining by flow cytometry. (G) The protein expression of p-AMPK (T172) and AMPK of HT-29 cells. (H) The protein expression of PGC-1α, TFAM and VDAC of HT-29 cells. (I) The protein expression of ZO-1, occludin, claudin-1, and claudin-3 of HT-29 cells. (J) The protein expression of p-MLC2, MLC2, and MLCK of HT-29 cells. (K) The TER value of the HT-29 cell (n = 6). Data is presented as the mean ± SEM. *P < 0.05; **P < 0.01; ***P < 0.001.

To further investigate the impact of 5-HT on mitochondrial biogenesis in the colon, we conducted *in vitro* experiments using HT-29 cells. Stimulation with 5-HT led to increased mitochondrial fragmentation (Fig. 5A). Additionally, 5-HT treatment resulted in a decrease in ATP and mtDNA levels (Fig. 5B, C), as well as a reduction in mitochondrial membrane potential (Fig. 5D). Moreover, 5-HT treatment increased levels of intracellular reactive oxygen species (ROS) and mitochondrial ROS (mtROS) (Fig. 5E, F). Treatment with MT effectively reversed these effects, indicating that 5-HT disrupts the structure and function of intestinal epithelial cell mitochondria via oxidative stress pathways. At the protein level, 5-HT treated groups exhibited reduced levels of p-AMPK (T172) (Fig. 5G). The expression of PGC-1α, TFAM, and VDAC also decreased (Fig. 5H), suggesting an inhibition of the pathways that regulate mitochondrial biogenesis. MT treatment ameliorated the inhibitory effects of 5-HT on AMPK phosphorylation and mitochondrial biogenesis-related proteins. To validate the general effect of 5-HT on intestinal epithelial cells, we conducted tests on mitochondrial biogenesis-related proteins using Caco-2 cells. The results were consistent with those observed in HT-29 cells, 5-HT treatment suppressed AMPK phosphorylation levels and reduced the expression of PGC-1α and TFAM, while MT treatment mitigated these effects (Fig. S3A, B). Remarkably, HT-29 cells treated with 5-HT expressed lower levels of tight junction proteins, including ZO-1, occludin, claudin-1, and claudin-3 (Fig. 5I). Importantly, MT intervention significantly mitigated the reduction in tight junction protein expression caused by 5-HT (Fig. 5I, J), which was further confirmed by TER measurements, suggesting that MT partially reversed the barrier function reduction caused by 5-HT (Fig. 5K). reinforcing our hypothesis that 5-HT disrupts the tight junction integrity by affecting mitochondrial biogenesis.

### Serotonin impairs mitochondrial biogenesis through inhibition of AMPK phosphorylation

**Figure 6:**
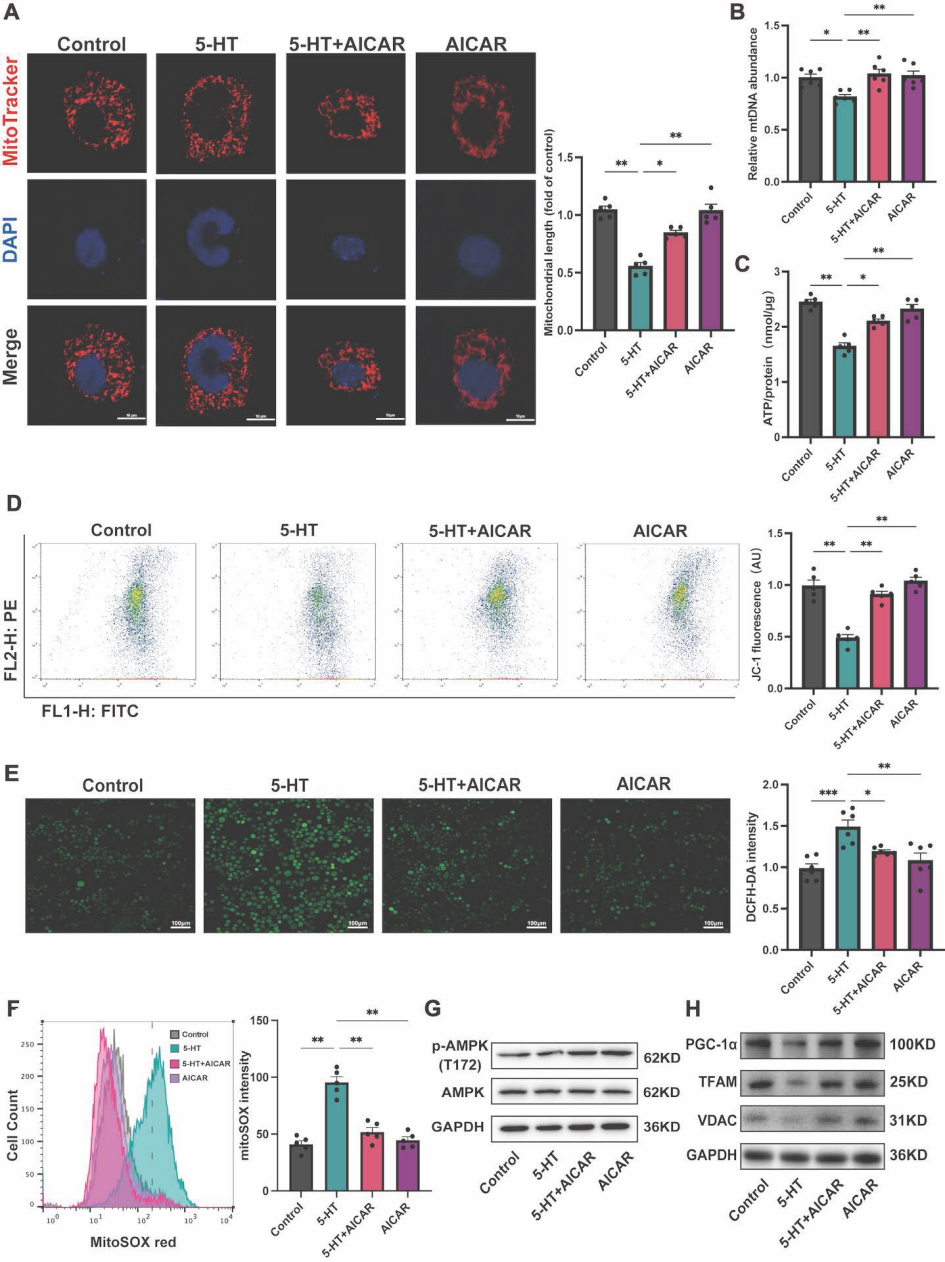
** 5-HT Inhibits AMPK Phosphorylation to Disrupt Mitochondria.** (A) Representative micrographs showing MitoTracker staining in HT-29 cells treated with AICAR (250 μM) for 1 h followed by 5-HT treatment for 24 h (scale bar = 10 µm) and quantification of mitochondrial length. (B) Measurement of mtDNA levels of HT-29 cells (n = 6). (C) Measurement of ATP levels of HT-29 cells (n = 5). (D) Measurement of mitochondrial membrane potential by JC-1 probes. (E) Determination of ROS levels in HT-29 cells by DCFH-DA fluorescent staining. (F) Measurement of mitochondrial ROS using MitoSOX staining by flow cytometry. (G) The protein expression of p-AMPK (T172) and AMPK of HT-29 cells. (H) The protein expression of PGC-1α, TFAM and VDAC of HT-29 cells. Data is presented as the mean ± SEM. *P < 0.05; **P < 0.01; ***P < 0.001.

To elucidate the effect of 5-HT on the AMPK-PGC-1α signaling pathway, we treated HT-29 cells with AICAR, an AMPK phosphorylation activator. MitoTracker staining revealed that AICAR ameliorated the increase in mitochondrial fragmentation caused by 5-HT treatment (Fig. 6A) and inhibited the reduction in ATP production (Fig. 6B) and mtDNA content (Fig. 6C). Furthermore, AICAR improved the reduction in mitochondrial membrane potential in 5-HT treated cells (Fig. 6D) and suppressed the increase in mtROS production (Fig. 6E) and cellular ROS levels (Fig. 6F) after 5-HT exposure. At the protein level, AICAR reversed the inhibition of p-AMPK(T172) in 5-HT treated cells(Fig. 6G). The expression of PGC-1α, TFAM and VDAC in HT-29 and Caco-2 cells also increased after AICAR treatment (Fig. 6H, S4A). Collectively, these results collectively indicate that 5-HT interferes with mitochondrial biogenesis in intestinal epithelial cell by inhibiting AMPK phosphorylation, and the activation of AMPK by AICAR alleviated the adverse effects of 5-HT, reaffirming the role of the AMPK-PGC-1α axis in maintaining mitochondrial function.

**Figure 7:**
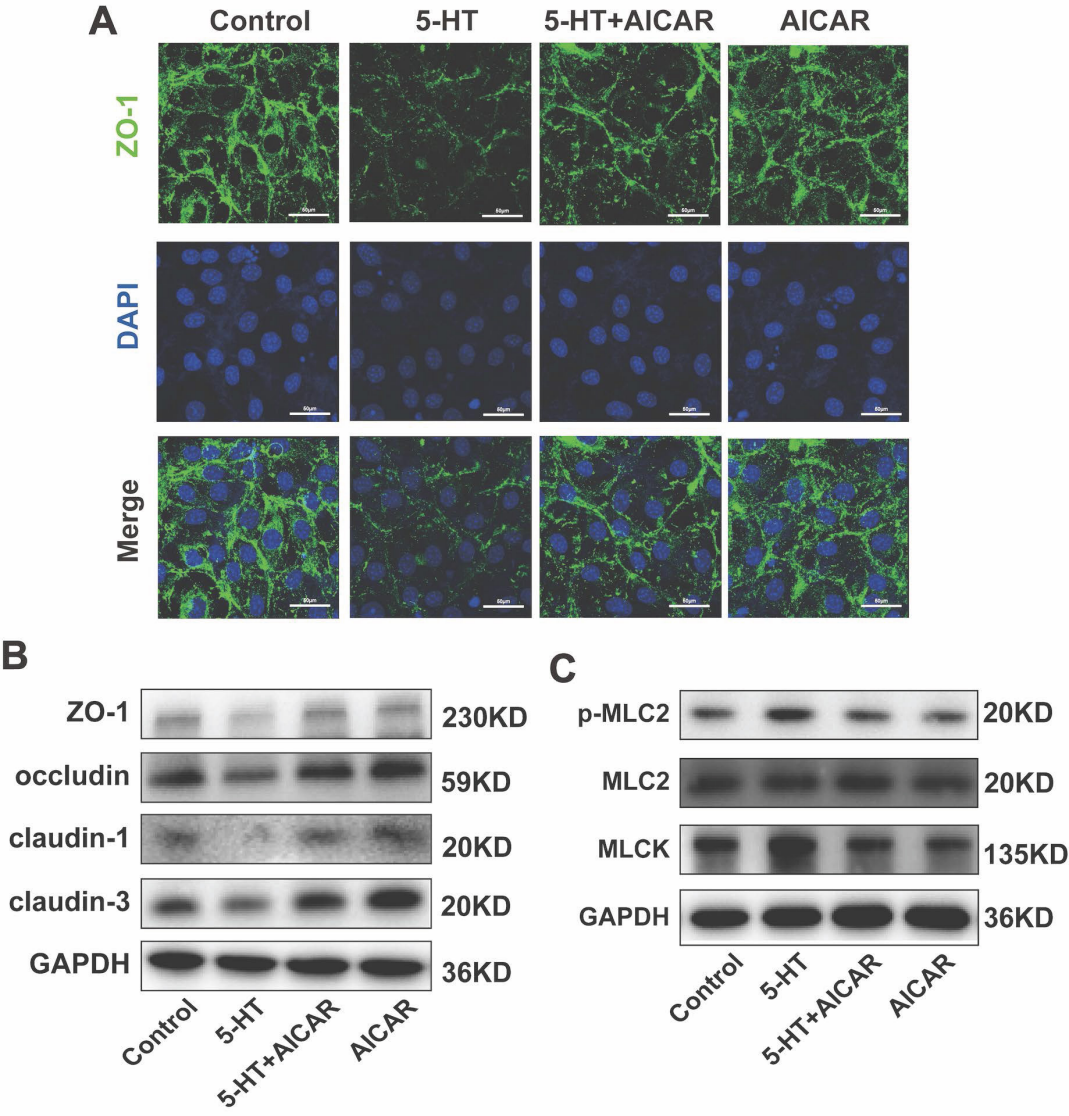
** AMPK Activation Inhibits the Reduction of Tight Junction Proteins Caused by 5-HT.** (A) Immunofluorescence staining for ZO-1 in HT-29 cells treated with AICAR (250 μM) for 1 h followed by 5-HT treatment for 24 h (scale bar = 50 µm). (B) The protein expression of ZO-1, occludin, claudin-1, and claudin-3 of HT-29 cells. (C) The protein expression of p-MLC2, MLC2, and MLCK of HT-29 cells.

Furthermore, AICAR treatment restored the reduction in tight junction protein expression caused by 5-HT (Fig. 7A, B, S4B) and countered the increase in phosphorylated p-MLC2 and MLCK induced by 5-HT (Fig. 7D, S4C). These results suggest that AMPK activation can prevent 5-HT-mediated disruption of tight junctions and related cellular stress responses.

### 5-HT_7_ receptor mediates 5-HT-induced inhibition of mitochondrial biogenesis via the cAMP-PKA pathway

**Figure 8:**
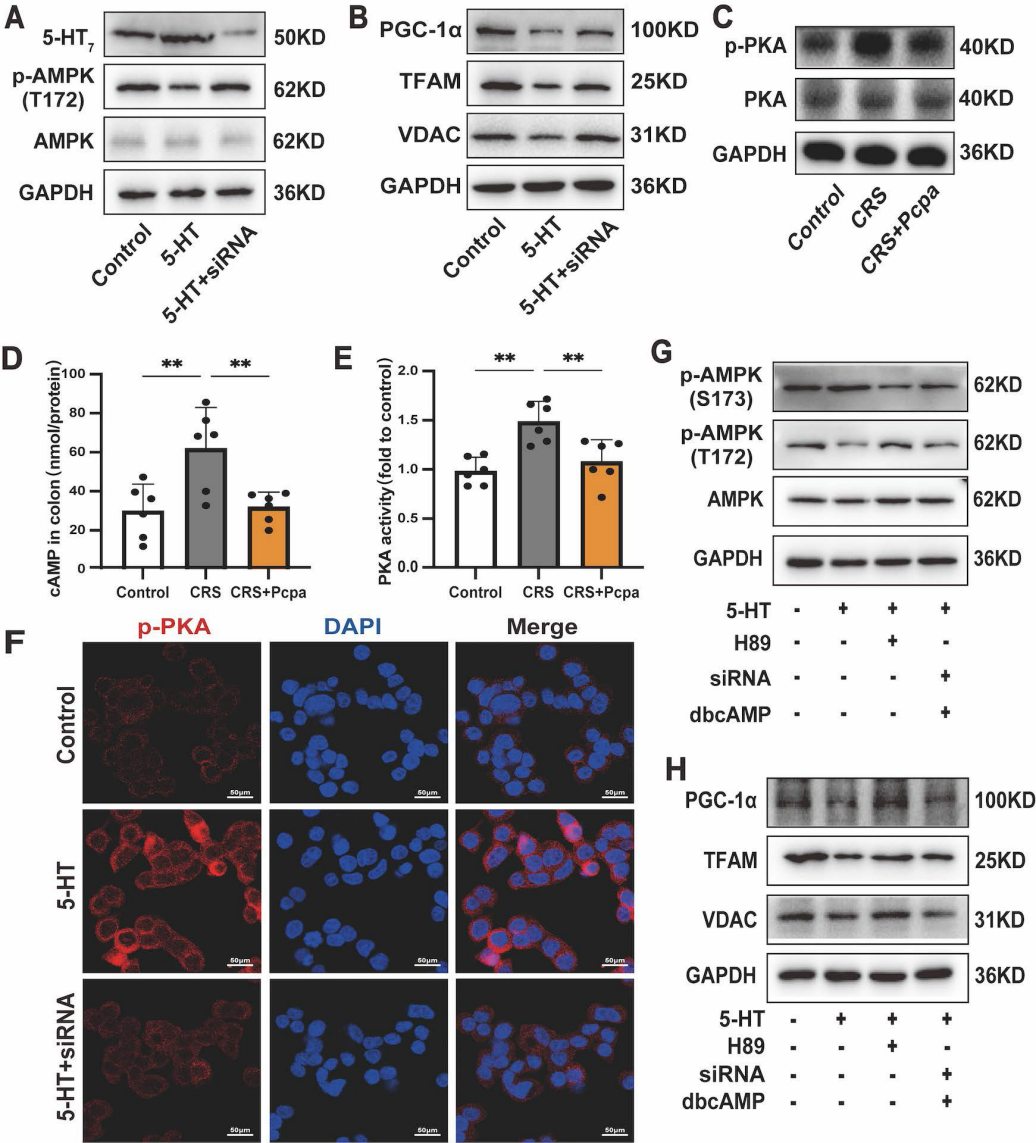
** 5-HT_7_/cAMP-PKA Mediates the Damaging Effects of 5-HT on Mitochondrial Biogenesis.** (A) The protein expression of 5-HT_7_ receptors, p-AMPK (T172) and AMPK of HT-29 cells treated with 5-HT (10 μM) for 24 h. The HT-29 cells were transfected with control siRNA or si-HTR7 (siRNA). (B) The protein expression of PGC-1α, TFAM and VDAC of HT-29 cells. (C) The protein expression of p-PKA, and PKA in the colon of mice. (D) The cAMP levels in the colon of mice (n = 6). (E) The PKA activity in the colon of mice (n = 6). (F) Immunofluorescence staining for p-PKA in HT-29 cells. (G) The protein expression of p-AMPK (S173), p-AMPK (T172) and AMPK of HT-29 cells treated with 5-HT, either alone or in combination with H-89 (1 μM) and dbcAMP (1 μM). The HT-29 cells were transfected with control siRNA or 5-HT_7_ siRNA (siRNA). (H) The protein expression of PGC-1α, TFAM and VDAC of HT-29 cells. Control: control group; CRS: chronic restraint stress group; CRS+Pcpa: chronic restraint stress + Pcpa intervention group. Data is presented as the mean ± SEM. *P < 0.05; **P < 0.01.

Given the role of 5-HT in epithelial cells requires its receptor interactions^36^, we sought to elucidate the specific pathways through which 5-HT inhibits AMPK phosphorylation. Initially, we evaluated the expression levels of 5-HT receptors in the intestines of mice subjected to chronic stress. The results revealed that the expressions of 5-HT_4_ and 5-HT_7_ receptors in the colon of CRS mice were significantly up-regulated and subsequently down-regulated following Pcpa treatment (Fig. S5A, B). Consequently, we treated HT-29 cells with the 5-HT_4_ receptor antagonist GR113808 and the 5-HT_7_ receptor antagonist SB269970. The results demonstrated that while the 5-HT_4_ antagonist mitigated the inhibition of AMPK phosphorylation by 5-HT to a lesser extent, the application of the 5-HT_7_ antagonist SB269970 essentially reversed the 5-HT-induced reduction in AMPK phosphorylation (Fig. S5C). Moreover, SB269970 significantly attenuated the 5-HT-induced down-regulation of PGC-1α, TFAM, and VDAC induced by 5-HT (Fig. S5D). Fluorescence findings provided additional confirmation of the 5-HT_7_ receptor's response to 5-HT in the colon of CRS mice (Fig. S5E). To confirm the role of the 5-HT_7_ receptor in mitochondrial biogenesis, we generated 5-HT_7_ knockdown HT-29 cells using siRNA (Fig. 8A). Treatment with si-HTR7 restored the phosphorylation levels of AMPK in HT-29 cells exposed to 5-HT (Fig. 8A). Additionally, the expression of mitochondrial biogenesis-related proteins, including PGC-1α, TFAM, and VDAC, was also restored (Fig. 8B). These results indicate that the 5-HT_7_ receptor plays a major role in the 5-HT-induced impairment of mitochondrial biogenesis in intestinal epithelial cells.

Subsequent studies focused on the downstream signaling of the 5-HT_7_ receptor. As cAMP is a downstream messenger of 5-HT_7_^37^, we sought to determine whether the cAMP-PKA pathway was implicated in the 5-HT_7_ receptor-mediated effects by assessing the levels of phosphorylated PKA. The results indicated that phosphorylated PKA was elevated in CRS mice and returned to levels comparable to the control group following Pcpa treatment (Fig. 8C). Furthermore, the assessment of cAMP and PKA activity in the mouse colon (Fig. 8D, E) provided evidence that 5-HT mediated the activation of the cAMP-PKA pathway in CRS mice. Additionally, 5-HT significantly increased PKA phosphorylation in HT-29 cells, while si-HTR7 inhibited this increase (Fig. 8F).

To further substantiate the involvement of the cAMP-PKA pathway, we utilized the PKA inhibitor H-89 and the cAMP analog dbcAMP. Previous studies have shown that PKA inhibits AMPK activation by associating with Ser-173, thereby preventing the phosphorylation of threonine (Thr-172)^38^. Our results indicated that in HT-29 cells exposed to 5-HT, the levels of p-AMPK(S173) increased while p-AMPK(T172) decreased. The PKA phosphorylation inhibitor H-89 reversed this effect (Fig. 8G). Conversely, in the presence of dbcAMP, the ability of si-HTR7 to rescue p-AMPK(T172) was lost, accompanied by elevated levels of p-AMPK(S173). This demonstrates that PKA mediates the inhibitory effect of the 5-HT/5-HT_7_ receptor on AMPK activation (Fig. 8G).

Moreover, inhibiting PKA phosphorylation mitigated the 5-HT-induced suppression of mitochondrial biogenesis in HT-29 cells, restoring the levels of PGC-1α, TFAM, and VDAC (Fig. 8H). However, in the presence of dbcAMP, si-HTR7 could not reverse the 5-HT-induced downregulation of PGC-1α, TFAM, and VDAC (Fig. 8H). dbcAMP treatment also abolished the beneficial effects of the 5-HT_7_ receptor antagonist SB269970 on mitochondrial biogenesis (Fig. S5F, G). In conclusion, the 5-HT_7_ receptor is crucial in 5-HT-induced damage in intestinal epithelial cells, acting through the cAMP-PKA pathway to reduce AMPK phosphorylation and inhibit mitochondrial biogenesis.

### Antagonizing 5-HT_7_ alleviates intestinal barrier damage induced by chronic stress

**Figure 9:**
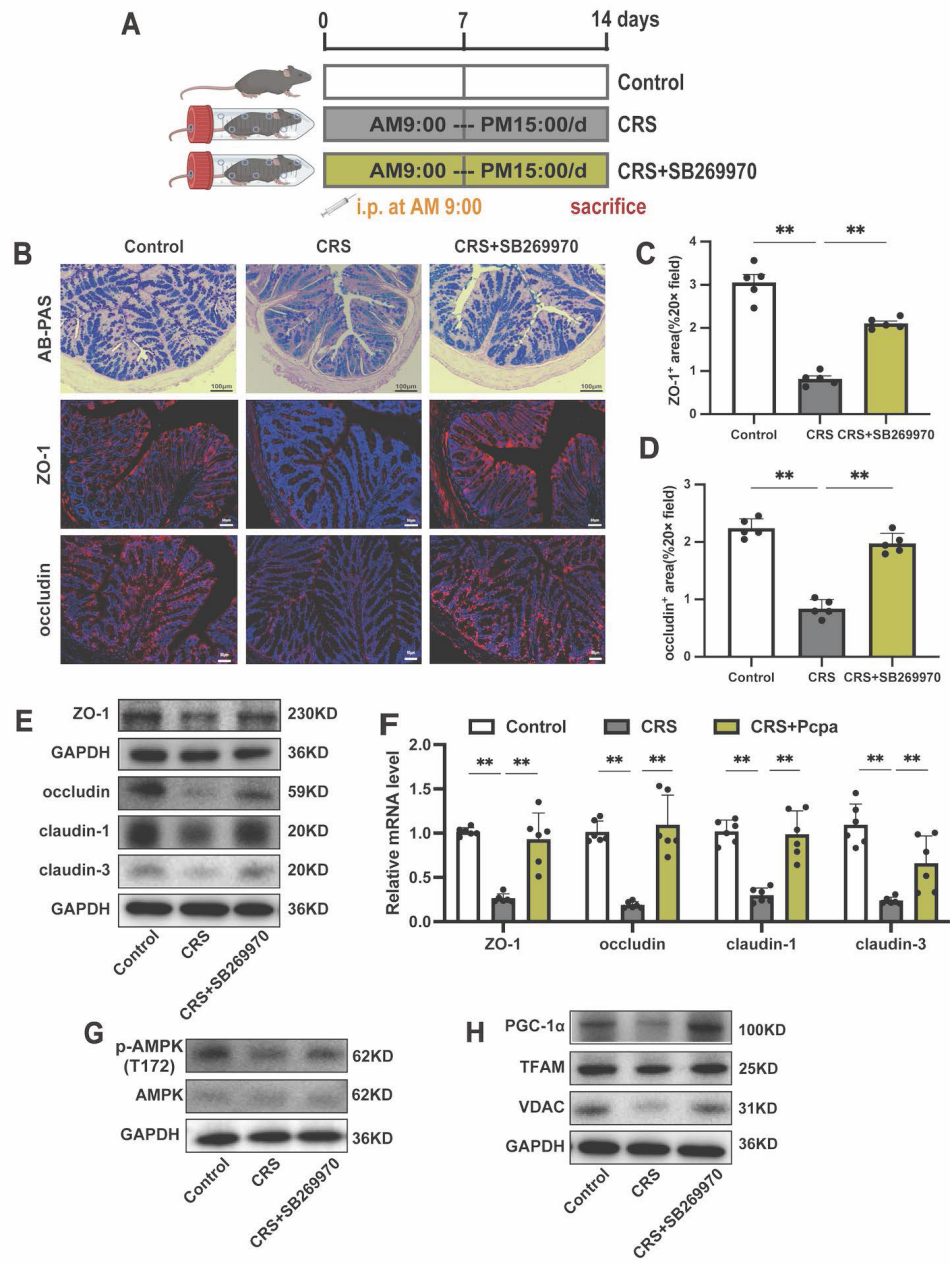
** Inhibiting 5HT_7_ Alleviates Intestinal Damage in Chronically Stressed Mice.** (A) Intervention and grouping schematic for SB269970. (B) AB-PAS staining (scale bar = 100 µm), ZO-1 IF staining (scale bar = 50 µm), and occludin IF staining (scale bar = 50 µm) in the colon of mice. Quantitative analysis of ZO-1 expression (C) and occludin expression (D). (E) The protein expression of ZO-1, occludin, claudin-1, and claudin-3 in the colon. (F) The mRNA levels of ZO-1, occludin, claudin-1, and claudin-3 in the colon (n = 6). (G)The protein expression of p-AMPK (T172) and AMPK in the colon. (H) The protein expression of PGC-1α, TFAM and VDAC in the colon. Control: control group; CRS: chronic restraint stress group; CRS+SB269970: chronic restraint stress + SB269970 intervention group. Data is presented as the mean ± SEM. *P < 0.05; **P < 0.01.

Finally, we aimed to validate the *in vivo* pharmacological effects of a 5-HT_7_ antagonist in counteracting the chronic stress-induced intestinal damage (Fig. 9A). AB-PAS staining revealed that the 5-HT_7_ antagonist rescued the reduction in goblet cells in the colon of CRS mice (Fig. 9B). Following treatment with the 5-HT_7_ receptor antagonist SB269970, the significant downregulation of tight junction proteins in CRS mice was reversed (Fig. 9B-F). Furthermore, we investigated the effect of SB269970 on the phosphorylation of AMPK and found that this treatment alleviated the reduction in p-AMPK (T172) levels in the colon of CRS mice, as well as the expression of PGC-1α, TFAM, and VDAC (Fig. 9G, H). These findings suggest that the 5-HT_7_ antagonist not only protects tight junctions but also supports mitochondrial function under chronic stress conditions. In summary, the results from our mouse model confirm that the 5-HT_7_ antagonist SB269970 could pharmacologically alleviate the adverse effects of CRS on the intestinal barrier.

## Discussion

Chronic stress-induced dysregulation of the brain-gut axis is a common etiological factor in gastrointestinal diseases, leading to impaired intestinal barrier function and exacerbated disease symptoms^7^,^39^. The present study provides substantial evidence revealing the pathway through which 5-HT, a neurotransmitter well-known for its role in the central nervous system, disrupts the intestinal barrier under stress conditions. Specifically, 5-HT acts as a signaling molecule in response to stress stimuli, impairing mitochondrial function in intestinal epithelial cells, ultimately intestinal barrier damage. These findings elucidate the impact of the neurotransmitter 5-HT on the intestine under chronic stress and offer insights for the development of treatment strategies for stress-related gastrointestinal disorders.

In the intestine, ECs synthesize more than 90% of the body's 5-HT through TPH1 and inactivate it under the action of SERT to maintain the physiological homeostasis of 5-HT in the intestine^40^. Our results demonstrate that chronic stress-induced elevation of intestinal 5-HT levels is associated with alterations in the expression of biomarkers related to 5-HT synthesis and degradation. We observed upregulation of TPH1 and AADC, along with downregulation of MAOA and SERT. The synergistic changes in 5-HT metabolism may contribute to the accumulation of 5-HT in the intestine under chronic stress conditions^41^. Consistently, in gastrointestinal diseases such as IBS and IBD, dysregulation of TPH1 and SERT expression has been repeatedly reported, further reinforcing the correlation between chronic stress-induced changes in 5-HT induced by chronic stress and the susceptibility to gastrointestinal disorders.

The intestinal epithelium, composed of various cell types including absorptive enterocytes, goblet cells, Paneth cells, enteroendocrine cells, and stem cells^42^, exhibits metabolic adaptability in response to internal and external signals. It dynamically adjusts its structure and function in response to fluctuating environmental factors to maintain intestinal health^43^,^44^. Chronic stress can disrupt neurotransmitter and metabolic profiles through the gut-brain axis, leading to damage to intestinal structure and integrity^45^. In this context, we observed a reduction in goblet cells and MUC2 expression in CRS mice, consistent with previous reports^39^,^46^. Our research shows that the number of goblet cells and mucin expression in the intestines of CRS mice are related to 5-HT levels. Previous studies have shown that in weaning mice with stress-induced diarrhea, 5-HT levels are elevated while IL-4 levels are significantly reduced^47^. IL-4, a key Th2 immune cytokine, can stimulate the production and activity of goblet cells, thereby enhancing the intestinal mucosal barrier^48^. Similarly, in CRS-induced colorectal cancer mice, increased 5-HT levels are accompanied by reduced Th2 cells and lower IL-4 levels^49^. These findings suggest that under stress conditions, 5-HT and IL-4 may be linked in regulating goblet cell activity and mucosal immunity. Furthermore, research has found that goblet cells are lost in the intestines of CRS mice, but this can be alleviated by activating the sympathetic nervous system^46^. Other studies have confirmed that cholinergic signaling is impaired in CRS models^50^. Mice subjected to repeated water avoidance stress (rWAS) showed increased acetylcholine transferase levels, indicating changes in cholinergic signaling^51^. Although there is no difference in the total amount of mucus in the distal colon of these mice, stress-related cholinergic changes may alter the nature of the mucus. In our study, whether changes in 5-HT levels are accompanied by a loss of cholinergic signaling has not yet been explored. Further investigation is needed to determine if 5-HT and cholinergic signaling jointly regulate goblet cell activity and mucus production.

Moreover, we found inhibited proliferation of the intestinal epithelium, increased intestinal permeability and fecal water content, intensified oxidative stress, and downregulation of tight junction proteins in CRS mice. Sabah Haq et al. demonstrated that supplementing 5-HT in DSS-induced colitis mice impairs autophagy in intestinal epithelial cells and increases the severity of intestinal inflammation^13^. Similarly, in our study, the use of Pcpa to inhibit 5-HT alleviated the changes in intestinal structure and function in CRS mice, confirming the detrimental effects of 5-HT on the intestine under certain conditions.

Healthy mitochondria are vital for cellular energy supply, and any disruption in mitochondrial function can affect overall cellular function and ultimately impact tissue and organ function.^19^. In the intestine, mitochondria maintain the intestinal epithelial barrier by regulating the integrity of tight junctions in epithelial cells and epithelial cell self-renewal^46^,^52^. Oxidative stress, a common consequence of chronic stress, can induce mitochondrial damage through the oxidation of proteins, lipids, and DNA^53^, and although ROS are important signaling intermediates involved in various homeostatic molecular pathways 54. Studies on the impaired integrity model of the Caco-2 intestinal epithelial cell monolayer have shown that mtROS are involved in the redistribution of occludin and ZO-1 from tight junctions to intracellular compartments, leading to structural changes in intercellular connections^55^. Excess ROS can oxidize and damage the actin and tubulin proteins of intestinal epithelial cells, altering the normal organization of the cytoskeleton and increasing paracellular permeability of the epithelium^56^. Our findings suggest that reducing the biosynthesis of 5-HT can inhibit colon mitochondrial damage in CRS mice and promote mitochondrial biogenesis. Consistently, the addition of 5-HT to HT-29 cells promoted mitochondrial damage and ROS generation while inhibiting the expression of tight junction proteins, highlighting the potential connection between 5-HT, mitochondrial damage, and intestinal barrier integrity. Mitochondrial-targeted antioxidants such as Mito-tempo (MT) can protect epithelial barrier function and reduce signs of oxidative stress in intestinal mucosal biopsy samples from CD patients. In our study, MT increased the expression of tight junction proteins in the colon of CRS mice and played a role in crucial intermediate events in tight junction regulation, specifically MLCK-dependent phosphorylation of MLC2. Pretreatment with MT inhibited the changes in tight junction proteins and the MLCK-MLC pathway induced by serotonin in HT-29 cells. This finding confirms that mitochondrial damage is an intermediary pathway through which serotonin disrupts intestinal barrier function. Mitochondrial homeostasis is primarily determined by the balance between mitochondrial autophagy, mitochondrial biogenesis, and the companion-mediated mitochondrial unfolded protein response^57^. Our results also observed a reduction in autophagy in the colons of CRS mice and impaired mitophagy pathways. Inhibiting 5-HT synthesis effectively improved this condition, highlighting the multiple effects of 5-HT on mitochondrial function. Although previous studies have reported the interaction between 5-HT signaling and autophagy in the colon^13^, the specific mechanisms by which 5-HT regulates mitophagy remain unclear and warrant further investigation.

PGC-1α, a master regulator of mitochondrial biogenesis, is involved in the transcriptional control of TFAM, which participates in mtDNA transcription and replication, thus maintaining mitochondrial homeostasis^58^. AMPK, a crucial energy sensor in cells, has been recently implicated in regulating mitochondrial biogenesis through the AMPK-PGC-1α signaling pathway 59. In intestinal epithelial cells, AMPK and PGC-1α have been shown to participate in feedback mechanisms that stabilize tight junctions and prevent stress-induced epithelial barrier dysfunction^60^-^62^. In our study, we observed inhibited AMPK phosphorylation in CRS mice and 5-HT treated intestinal epithelial cells. This observation is corroborated by a recent study which reported that elevated colonic 5-HT levels are associated with decreased AMPKα phosphorylation^13^. Moreover, Guragain et al. confirmed that 5-HT treatment of colon epithelial cells induces AMPKα dephosphorylation in a dose-dependent manner^63^. Our results also demonstrated that 5-HT caused a decrease in the expression of downstream mitochondrial biogenesis proteins PGC-1α and TFAM. However, treatment with AICAR (an AMPK phosphorylation activator) mitigated the detrimental effects of 5-HT, further implying that 5-HT's regulatory impact on colon epithelial cell. PGC-1α and mitochondrial function may primarily attributed to 5-HT-induced AMPK dephosphorylation. While previous studies have reported the inhibitory effect of 5-HT on AMPK, our findings provide strong evidence suggesting that elevated 5-HT levels may mediate their impact on the intestinal barrier through the disruption of mitochondrial biogenesis associated with the AMPK-PGC-1α signaling pathway.

5-HT exerts multiple effects on the intestine mediated by different receptors.^64^. Previous results by Sabah et al. suggested that 5-HT disrupts autophagy via the 5-HT_3_, 5-HT_4_, and 5-HT_7_ receptors, increasing susceptibility to experimental colitis and CD in mice^13^. Another study by the same group indicated that blocking 5-HT_7_ receptor signaling or genetic deletion of this receptor could alleviate the severity of chemically induced colitis^30^. Furthermore, antagonists of 5-HT_3_, 5-HT_4_, and 5-HT_7_ significantly inhibited 5-HT-induced monocyte-colon epithelial cell adhesion^15^. Studies have also shown that the 5HT_3_ receptor enhances NLRP3-mediated inflammation in colon macrophages, promoting the progression of colorectal cancer^14^. Our research demonstrate significant changes in the expression of 5-HT_4_ and 5HT_7_ receptors in CRS mice. Moreover, using the 5-HT_7_ receptor antagonist SB269970 or knocking down the 5-HT_7_ receptor with si-HTR7 in HT-29 cells effectively inhibited 5-HT-induced AMPK dephosphorylation. This also mitigated the adverse effects of 5-HT on mitochondrial biogenesis and tight junction protein expression. Notably, we established a link between the second messenger cAMP^65^, associated with the 5-HT_7_ receptor, and p-AMPK (T172). Previous findings have shown that the cAMP/PKA signaling pathway responds to chronic stress^8^. In 5-HT-treated HT-29 cells, activation of the cAMP-PKA pathway nullified the beneficial effects of inhibiting the 5-HT_7_ receptor on mitochondrial biogenesis, indicating that the actions mediated by the 5-HT_7_ receptor depend on this signaling cascade. Prior research have shown that, in mammals, AMPK phosphorylation at Ser173 by PKA antagonizes the activating phosphorylation at Thr172 by the LKB1 kinase^38^. Additionally, serotonin regulates feeding behavior in *C. elegans* through PKA-mediated phosphorylation of AMPK at Ser244 (equivalent to mammalian Ser173)^66^. Our results also demonstrated that 5-HT induces an increase in p-AMPK(S173) and a decrease in p-AMPK(T172). These findings provide robust evidence supporting the existence of the 5-HT/5-HT_7_-PKA-AMPK signaling cascade.

Lastly, administration of the 5-HT_7_ receptor antagonist SB269970 increased the expression of tight junction proteins and proteins related to mitochondrial biogenesis in the colon of mice subjected to CRS. This observation revealed the *in vivo* pharmacological effects of the 5-HT_7_ receptor antagonist in alleviating chronic stress-induced intestinal barrier damage and highlighted the potential of targeting 5-HT_7_ as a therapeutic approach for stress-related intestinal pathologies. Collectively, our results underscore the broader impacts of the neurotransmitter 5-HT beyond its traditional neural roles, suggesting that lowering 5-HT levels or blocking its receptors, particularly 5-HT_7_, may confer significant benefits in maintaining intestinal barrier integrity and function during periods of chronic stress.

## Conclusion

In conclusion, our findings substantiate the detrimental impact of 5-HT on the intestinal barrier during chronic stress. Elevated 5-HT levels exacerbate oxidative stress and disrupt mitochondrial biogenesis in intestinal epithelial cells, leading to decreased expression of tight junction proteins. Activation of the cAMP-PKA signaling cascade via 5-HT_7_ receptors on intestinal epithelial cells induces AMPK dephosphorylation, thereby hindering mitochondrial biogenesis and causing a dysregulation in the expression of tight junction proteins.

## Supplementary Material

Supplementary figures and tables.

## Abbreviations

AADC: aromatic l-amino acid decarboxylase
AMPK: AMP-activated protein kinase
CAT: Catalase
CD: Crohn's disease
CgA: chromogranin A
CORT: cortisol
CRS: chronic restraint stress
ECs: enterochromaffin cells
GSH/GSSG: Glutathione/oxidized Glutathione ratio
HPA: hypothalamic-pituitary-adrenal
IBD: inflammatory bowel disease
IBS: irritable bowel syndrome
MAOA: monoamine oxidase A
MDA: Malondialdehyde
MLC2: myosin light chain 2
MLCK: myosin light chain kinase
mtDNA: mitochondrial DNA
mtROS: mitochondrial ROS
NE: norepinephrine
OXPHOS: oxidative phosphorylation
PGC-1α: peroxisome proliferator-activated receptor gamma coactivator 1-alpha
qPCR: Quantitative PCR
rWAS: repeated water avoidance stress
ROS: reactive oxygen species
SERT: serotonin reuptake transporter
SOD: Superoxide Dismutase
T-AOC: Total Antioxidant Capacity
TER: transepithelial electrical resistance
TFAM: mitochondrial transcription factor A
TPH1: tryptophan hydroxylase 1
VDAC: Voltage-Dependent Anion Channels
HT: serotonin, 5-hydroxytryptamine)
8-OHdG: 8-hydroxy-2'-deoxyguanosine
