# Supplementary material for: Chronic Stress-induced Serotonin Impairs Intestinal Epithelial Cell Mitochondrial Biogenesis via the AMPK-PGC-1α Axis
Source: Int J Biol Sci. 2024 Aug 19;20(11):4476–95. doi: 10.7150/ijbs.97275 (PMC11380450; doi:10.7150/ijbs.97275)
Supplement: Supplementary file 1 — Supplementary figures and tables. [file ijbsv20p4476s1.pdf]

Supplementary Materials for

Chronic Stress-induced Serotonin Impairs Intestinal Epithelial Cell Mitochondrial Biogenesis via the AMPK-PGC-1α Axis

This PDF file includes:

Figs. S1 to S5

Other Supplementary Materials for this manuscript include the following:

Table S1 to S2

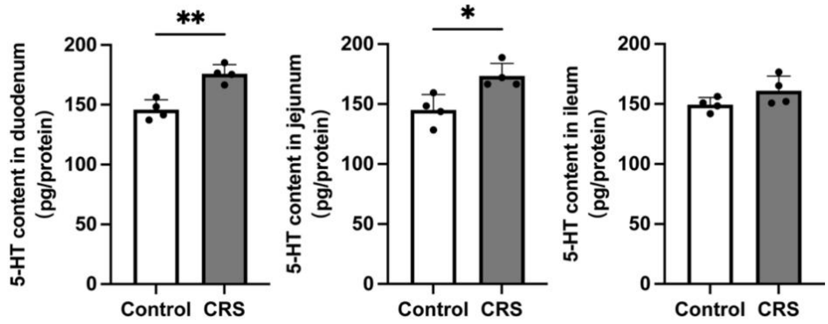

Figure S1. The effect of chronic restraint stress on the levels of 5-HT in the intestines of mice.

(A) 5-HT levels in duodenum (n = 4). (B) 5-HT levels in jejunum (n = 4). (C) 5-HT levels in ileum (n = 4). Data is presented as the mean ± SEM. *$P < 0.05$; **$P < 0.01$.

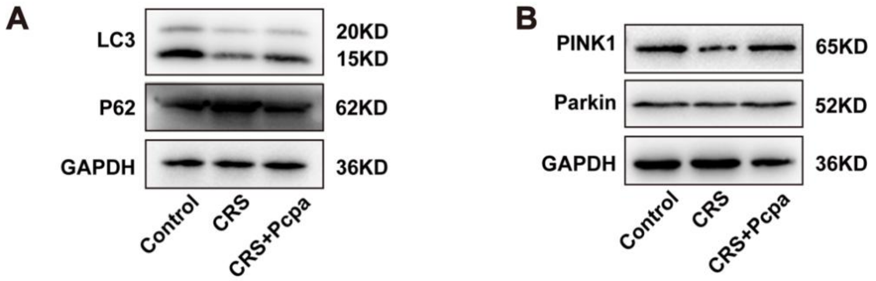

Figure S2. Inhibition of 5-HT alleviates colonic autophagy in CRS mice

(A) The protein expression level of LC3 and p62 in the colon. (B) The protein expression level of PINK1 and Parkin in the colon.

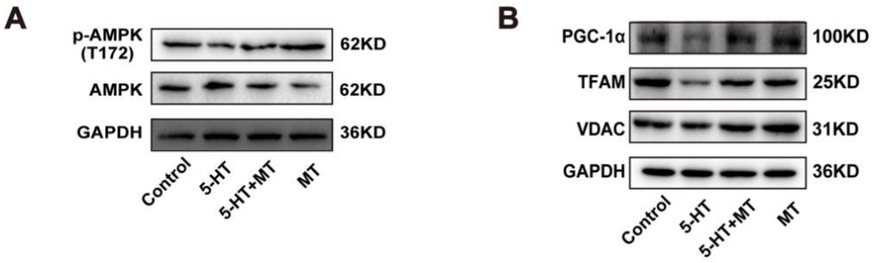

Figure S3. The effect of 5-HT on mitochondrial biogenesis-related proteins in Caco-2 cells.

(A) The protein expression of p-AMPK (T172) and AMPK of Caco-2 cells treated with 5-HT (10 μM) and Mito-tempo (MT, 10 μM) for 24 h. (B) The protein expression of PGC-1α, TFAM and VDAC of Caco-2 cells.

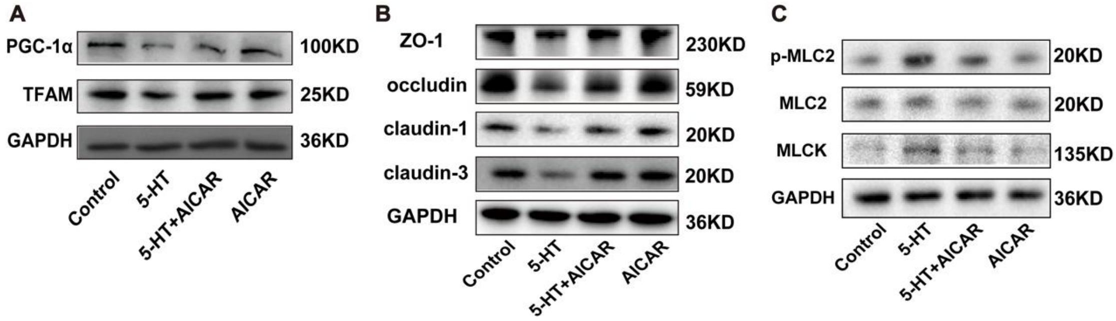

Figure S4. Activation of AMPK inhibits the reduction of tight junction proteins in Caco-2 cells caused by 5-HT.

(A) The protein expression of PGC-1α, TFAM and VDAC of Caco-2 cells treated with AICAR (250 μM) for 1 h followed by 5-HT treatment for 24 h. (B) The protein expression of ZO-1, occludin, claudin-1, and claudin-3 of Caco-2 cells. (C) The protein expression of p-MLC2, MLC2, and MLCK of Caco-2 cells.

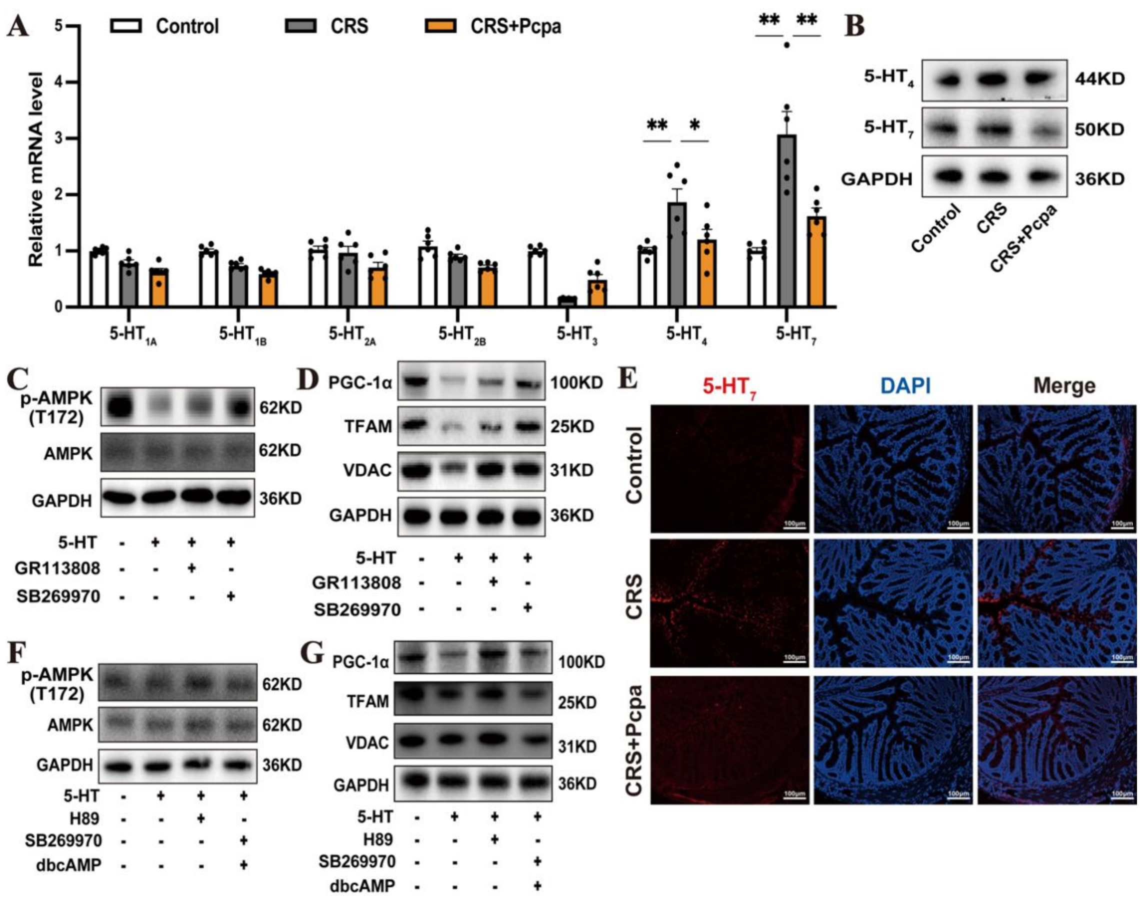

Figure S5. 5-HT regulates mitochondrial biogenesis in intestinal epithelial cells through 5-HT$_7$/PKA.

(A) The mRNA levels of 5-HT receptors in the colon (n = 6). (B) The protein expression of 5-HT4 and 5-HT7 receptors in the colon of mice. (C) The protein expression of p-AMPK (T172) and AMPK of HT-29 cells treated with GR113808 (10 nM) or SB269970 (10 μM) for 1 h followed by 5-HT treatment for 24 h. (D) The protein expression of PGC-1α, TFAM, and VDAC of HT-29 cells treated with GR113808 or SB269970 for 1 h followed by 5-HT treatment for 24 h. (E)Immunofluorescence staining of 5-HT$_7$ receptors in the colon (scale bar = 100μm). (F) The protein expression of p-AMPK (T172) and AMPK of HT-29 cells treated with 5-HT, either alone or in combination with H-89 (1 μM), dbcAMP (1 μM) and

SB269970. (G) The protein expression of PGC-1α, TFAM, and VDAC of HT-29 cells treated with 5-HT, H-89, dbcAMP, and SB269970. Data is presented as the mean ± SEM. *$P < 0.05$; **$P < 0.01$.

Table S1. Primers for real-time PCR

Gene name	Forward sequences (5'-3')	Reverse sequences (5'-3')
SERT	GTTGATGCTGCGGCTCAGATCT	GAAGCTCGTCATGCAGTTCACC
TPH1	TGTTGACTGCGACATCAGCCGA	GGAAACCAAGGGACAGTCTCCA
AADC	GGAGCCAGAAACATACGAGGAC	GCATGTCTGCAAGCATAGCTGG
MAOA	GGCTGTCATCAAGTGCATGGTG	GCAGGCATTGACCCATCTGGTT
ZO-1	GTTGGTACGGTGCCCTGAAAGA	GCTGACAGGTAGGACAGACGAT
occludin	TGGCAAGCGATCATACCCAGAG	CTGCCTGAAGTCATCCACACTC
claudin1	GGACTGTGGATGTCCTGCGTTT	GCCAATTACCATCAAGGCTCGG
claudin3	TCATCGTGGTGTCCATCCTGCT	AGAGCCGCCAACAGGAAAAGCA
ATP5a-1	TGGTGAAGAGACTGACGGATGC	TCAAAGCGTGCTTGCCGTTGTC
PGC-1α	GAATCAAGCCACTACAGACACCG	CATCCCTCTTGAGCCTTTCGTG
mtND1	GGCTATATACAACTACGCAAAGG	GGTAGATGTGGCGGGTTTTAGG
GAPDH	AAGGTGACAGCAGTCGGTT	TGTGTGGACTTGGGAGAGG
12sRNA	ACCGCGGTCATACGATTAAC	CCCAGTTTGGGTCTTAGCTG
18sRNA	CATTCGAACGTCTGCCCTATC	CCTGCTGCCTTCCTTGGA
5-HT_{1A}	TGCCAACTATCTCATCGGCTCC	CAGAGTCCACTTGTTGAGCACC
5-HT_{1B}	TCACTGACCTGCTCGTGTCCAT	TATCCGACGACAGCCAGAAGTC
5-HT_{2A}	CCTGATGTCACTTGCCATAGCTG	CAGGTAAATCCAGACGGCACAG
5-HT_{2B}	TGTGATGCCGATTGCCCTCTTG	ATAGCGGTCCAGGGAAATGGCA
5-HT_3	CACACTCCTTCTGGGATACTCAG	GATGGTCTCAGCGAGGCTTATC
5-HT_4	CTGGGCTTATGGGGAGATGTTCT	GCTGGGGCCTGCTTTCAGAG
5-HT_7	TCATGACCCTGTGCGTGATCAG	GAGAAGCCAGACCGACAGAATC
β-actin	TTGCTGACAGGATGCAGAAG	ACATCTGCTGGAAGGTGGAC

Table S2. Antibody for western blot

Antigen	Host	Dilution	Cat number	Source
TPH1	rabbit	1:1000	ab52954	Abcam
SERT	rabbit	1:1000	19559-1-AP	Proteintech
ZO-1	rabbit	1:1000	21773-1-AP,	Proteintech
occludin	rabbit	1:1000	27260-1-AP	Abcam
claudin-1	rabbit	1:3000	ab15098	Abcam
claudin-3	rabbit	1:1000	ab15102	Abcam
p-MLC2	rabbit	1:1000	29504-1-AP	Proteintech
MLC2	rabbit	1:1000	15354-1-AP	Proteintech
MLCK	rabbit	1:1000	21642-1-AP	Proteintech
p-AMPK(T172)	rabbit	1:1000	2535	CST
p-AMPK(S173)	rabbit	1:1000	bs-5575R	Bioss
AMPK	rabbit	1:1000	5831	CST
PGC-1α	rabbit	1:1000	ab313559	Abcam
TFAM	rabbit	1:1000	ab176558	Abcam
VDAC	rabbit	1:1000	10866-1-AP	Proteintech
LC3	rabbit	1:1000	Ab232940	Abcam
p62	rabbit	1:1000	67824	CST
PINK1	rabbit	1:1000	23274-AP	Proteintech
Parkin	rabbit	1:1000	14060-AP	Proteintech
p-PKA	mouse	1:1000	sc-377575	Santa Cruz
PKA	mouse	1:1000	sc-137220	Santa Cruz
HTR_7	rabbit	1:1000	DF13323	Affinity Biosciences
HTR_4	rabbit	1:1000	21165-1-AP	Proteintech
GAPDH	mouse	1:10000	66004-1-lg	Proteintech
HRP-conjugated IgG	mouse	1:10000	SA00001-1	Proteintech
HRP-conjugated IgG	rabbit	1:10000	SA00001-2	Proteintech
